# Circular stable intronic RNAs possess distinct biological features and are deregulated in bladder cancer

**DOI:** 10.1093/narcan/zcad041

**Published:** 2023-08-07

**Authors:** Asta M Rasmussen, Trine Line H Okholm, Michael Knudsen, Søren Vang, Lars Dyrskjøt, Thomas B Hansen, Jakob S Pedersen

**Affiliations:** Department of Clinical Medicine, Aarhus University, Aarhus 8000, Denmark; Department of Molecular Medicine (MOMA), Aarhus University Hospital, Aarhus N 8200, Denmark; Bioinformatics Research Center (BiRC), Aarhus University, Aarhus 8000, Denmark; Departments of Otolaryngology-Head and Neck Surgery and Microbiology & Immunology, University of California, San Francisco, CA, USA; Department of Clinical Medicine, Aarhus University, Aarhus 8000, Denmark; Department of Molecular Medicine (MOMA), Aarhus University Hospital, Aarhus N 8200, Denmark; Department of Clinical Medicine, Aarhus University, Aarhus 8000, Denmark; Department of Molecular Medicine (MOMA), Aarhus University Hospital, Aarhus N 8200, Denmark; Department of Clinical Medicine, Aarhus University, Aarhus 8000, Denmark; Department of Molecular Medicine (MOMA), Aarhus University Hospital, Aarhus N 8200, Denmark; Department of Molecular Biology and Genetics (MBG), Aarhus University, Aarhus 8000, Denmark; Department of Clinical Medicine, Aarhus University, Aarhus 8000, Denmark; Department of Molecular Medicine (MOMA), Aarhus University Hospital, Aarhus N 8200, Denmark; Bioinformatics Research Center (BiRC), Aarhus University, Aarhus 8000, Denmark

## Abstract

Until recently, intronic lariats were regarded as short-lasting splicing byproducts with no apparent function; however, increasing evidence of stable derivatives suggests regulatory roles. Yet little is known about their characteristics, functions, distribution, and expression in healthy and tumor tissue. Here, we profiled and characterized circular stable intronic sequence RNAs (sisRNAs) using total RNA-Seq data from bladder cancer (BC; *n* = 457, UROMOL cohort), healthy tissue (*n* = 46), and fractionated cell lines (*n* = 5). We found that the recently-discovered full-length intronic circles and the stable lariats formed distinct subclasses, with a surprisingly high intronic circle fraction in BC (∼45%) compared to healthy tissues (0–20%). The stable lariats and their host introns were characterized by small transcript sizes, highly conserved BP regions, enriched BP motifs, and localization in multiple cell fractions. Additionally, circular sisRNAs showed tissue-specific expression patterns. We found nine circular sisRNAs as differentially expressed across early-stage BC patients with different prognoses, and sisHNRNPK expression correlated with progression-free survival. In conclusion, we identify distinguishing biological features of circular sisRNAs and point to specific candidates (incl. sisHNRNPK, sisWDR13 and sisMBNL1) that were highly expressed, had evolutionary conserved sequences, or had clinical correlations, which may facilitate future studies and further insights into their functional roles.

## Graphical Abstract

**Graphical Abstract:**
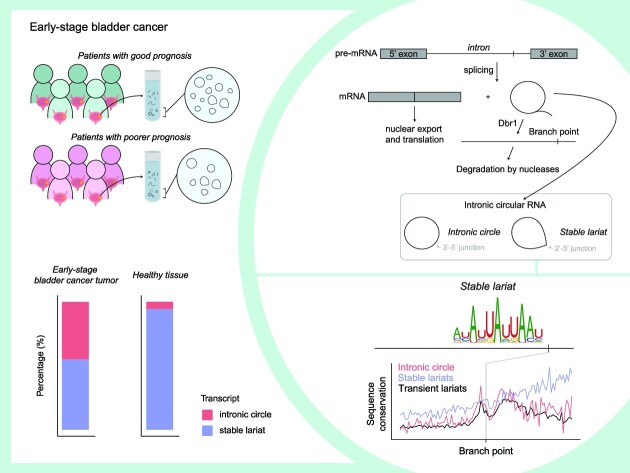

## INTRODUCTION

Although introns cover about half of the human genome, they are generally believed to be ephemeral short-lasting byproducts of RNA splicing (1). However, notable exceptions exist of stable functional molecules that are processed from excised lariats, e.g. microRNAs (miRNAs) and small nucleolar RNAs (snoRNAs) (2). Now, emerging evidence suggests that individual stable intronic RNAs persist in cells and possess functional roles of their own as linear or circular molecules.

Stable intron molecules are produced during canonical pre-mRNA splicing where the long non-coding intron elements are excised as branched RNA lariats. Two spliceosome-catalyzed transesterification events, involving the branch point (BP), 5’ splice site (5’ SS) and 3’ splice site (3’ SS), generate the intronic lariat byproduct containing the unique 2’-5’ phosphodiester linkage (3). The closed lariats are generated in an equal amount to the spliced exon junctions (mature mRNA) but are usually rapidly turned over. While most spliced mRNAs are transported to the cytoplasm, intronic lariats remain in the nucleus. Here, the rate-limiting step of lariat degradation is the Dbr1 induced hydrolysis of the 2’-5’ linkage. This leaves a linear debranched intronic RNA molecule, which is usually quickly degraded by exonucleases (4).

Stable lariats were first reported in T-cells (5) and viruses (6–9). Later, identification was made of intronic RNAs that accumulated in the oocyte nucleus of the frog *Xenopus tropicalis* and showed an unusual stability for up to two days, termed stable intronic sequence RNAs (sisRNAs) (10). While the nuclear sisRNAs exist as both lariats and linear molecules, sisRNAs in the cytoplasm of *Xenopus tropicalis* oocyte are only found in the form of circular sisRNAs (11); a class later described as comprising both stable lariats without tails, which escape debranching, and full-length intronic circles with a 3’-5’ linkage (12).

A complete circularization of the entire intron (3’-5’ circles) has also been observed in transcriptome-wide branch-point-mapping studies in human cells (13,14). These studies found that 3% of all inferred branch points mapped to the last intron position (3’ SS), creating full-length intronic circles. Intronic circles are suggested to originate from conventional lariats through a third nucleophilic attack on the 2’-5’ linkage or as a subsequent debranching-and-ligation reaction (13,14). While several intronic circles have been found (12,15) and validated (14,16,17), their stability, localization, and function have yet to be determined.

Several studies, across multiple species, have found that the majority of stable lariats (possessing the 2’-5’ branching junction) contain a cytosine (C) at their branch point instead of the canonical adenine (A) (12,18–20). Cytosine at the branch point may explain the resistance of stable lariats to the Dbr1 enzyme, which is inefficient at hydrolyzing the 2’-5’ linkage of C-branched lariats (21,22). Circular sisRNAs can also escape debranching through physical separation from Dbr1, which is achieved by export to the cytoplasm. Cytoplasmic circular sisRNAs have been identified in cells from zebrafish, frog, chicken, mouse, and human (12). The cytoplasmic stable lariats are generally derived from short introns (<500 bp), do not colocalize with their parent mRNA, and are exported to the cytoplasm via the NXF1/NXT1 machinery (12).

Though the majority of both linear and circular sisRNAs remain functionally uncharacterized, some are shown to have a specific role assigned to them (17,19,23–25). One example is circular molecules processed from lariats that escape debranching in human cell lines, termed circular intronic long non-coding RNAs (ciRNAs) (19). In particular, ci-ankrd52 associates with the polymerase II transcription machinery and positively regulates parent gene expression (19). However, the functional roles of circular sisRNAs in humans during health and disease are still largely unknown.

The role of non-coding RNA (ncRNA) in cell homeostasis is increasingly appreciated with a rising number of publications reporting ncRNA deregulation during a wide range of diseases (26–29). Cancer is a heterogeneous disease characterized by genomic instability and altered transcription patterns, with bladder cancer (BC) reported as the ninth most commonly occurring malignancy world-wide (30). Early stage BC (non-muscle invasive bladder cancer; NMIBC) is characterized by a low mortality rate but high recurrence rate leading to long-term patient-follow-up in the form of frequent and expensive cystoscopic monitoring, to avoid disease progression to a lethal muscle invasive stage (31,32). Discovering biomarkers that can help stratify tumors based on risk of progression is vital for patient-specific treatment and follow-up (33).

**Figure 1.**
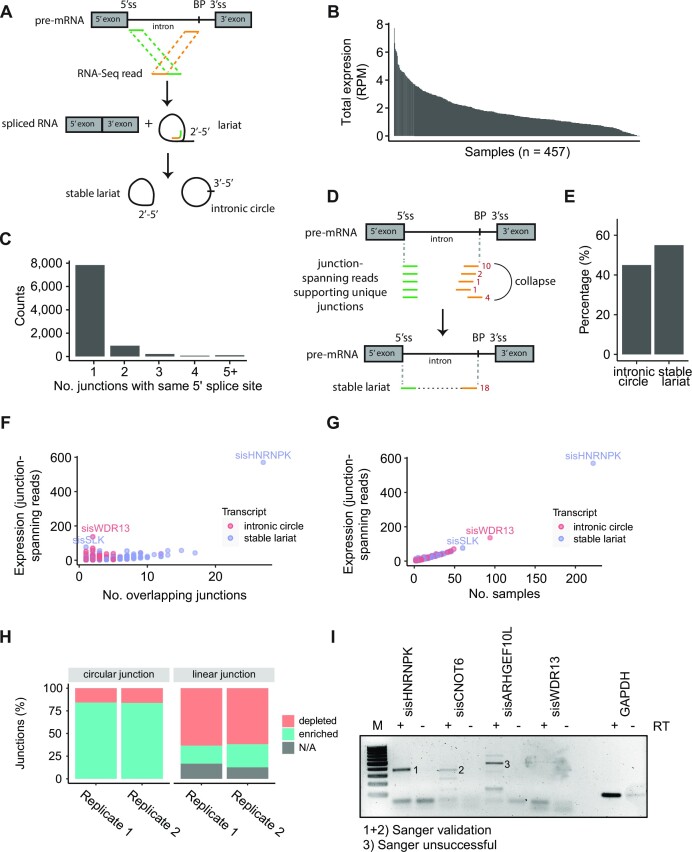
Identification and quantification of circular junctions. (**A**) Schematic overview of circular sisRNA biogenesis and identification. Circular sisRNAs are identified by an inverted read alignment strategy that identifies reads traversing the circularizing junction. Stable lariats escape debranching and are joined by a 2’-5’ bond, while circularized introns are joined by a 3’-5’ linkage and likely arise from a conventional lariat (1,2). (**B**) Total expression of junction-spanning reads (*n* = 11 420) per sample. RPM = reads per million. (**C**) Number of predicted junctions (*n* = 11 420) with the same 5’ splice site but with different branch point coordinates. (**D**) Predicted junctions, that are annotated to the same host intron and 5’ splice site but have different end coordinates, are collapsed using the annotation with the highest number of supporting junction-spanning reads. Counts (red) indicate reads supporting each junction. (**E**) Fraction of circular sisRNAs (*n* = 1827) that are defined as full-length circular introns (3’-5’ circles) or are identified as stable lariats (2’-5’ bond). (**F**) Cross-sample expression of circular sisRNAs (*n* = 1827) based on all collapsed junctions (see panel D) against the number of overlapping junctions. Colors indicate type of transcript. (**G**) Cross-sample expression of circular sisRNAs (*n* = 1827) plotted against the number of samples expressing the transcript. (**H**) The number of circular sisRNA junctions (left) and linear counterpart (right) that are enriched (blue) or unenriched (red) upon RNAse R treatment of HS68 cells (3). Only circular sisRNAs detected in both control and treated samples (>1 junction-spanning read) are included (*n* = 126 for replicate 1; *n* = 252 for Replicate 2). NA (grey) indicates that no linear counterpart is detected. (**I**) Validation of select circular sisRNAs in J28 cells by RT-PCR across the circularizing junction and subsequent Sanger sequencing. Successful Sanger validation is marked by numbers 1 and 2, and unsuccessful by 3. PCR amplification and Sanger sequencing were unsuccessful for sisWDR13.

Despite examples of individual sisRNAs, large-scale transcriptome-wide studies remain few as sisRNAs are absent from common data sets with polyA-selected mRNAs. Besides, exact identification of circular sisRNAs relies on precise alignments of the sequencing reads traversing the 5’ SS/BP splice junction (Figure 1A). While the reverse transcriptase (RT) is capable of reading through the nonconventional 2’-5’ bond, such events occur with low efficiency; and the RT is more likely to pause, fall off, or undergo template switching at this position resulting in mismatch errors, microinsertions, or deletions in the DNA product (34).

However, by analyzing sequenced total RNA from large sample collections, we were able to identify reads traversing the intronic lariat junction. Here, we identify and characterize circular sisRNAs in NMIBC. We show that they possess distinct biologic features potentially promoting stability, are present in multiple cellular compartments, have a tissue-specific expression pattern, comprise a highly conserved stable lariat subclass, and have an overall differential expression between prognostic risk classes in early-stage bladder cancer. This constitutes the first comprehensive analysis of circular sisRNAs in cancer.

## MATERIALS AND METHODS

### Identification and expression profiling of circular sisRNAs

The primary data set is a cohort of 457 non-muscle invasive bladder cancer (NMIBC) samples, which has been whole-transcriptome sequenced and clinically annotated in a previous study (35). We used STAR (36) to map the total RNA-Seq reads to the human reference genome hg38 (http://hgdownload.soe.ucsc.edu/downloads.html). Given the chimeric reads as input, we ran CIRCexplorer2 with default settings to detect circular sisRNAs and the number of circular-junction-supporting reads (37). The output from CIRCexplorer2 was compared with other circRNA detection and correction methods, namely, DCC (38), circRNA_Finder (39), and CIRIquant (40) (run on the full CIRCexplorer2 output) to investigate the level of overlap in their output. All pipelines were run with default settings. As only some methods provide elaborate annotation of whether a circRNA junction origin is exonic or intronic, we annotated all detected circular junctions using a non-exon-overlapping intron set.

Additionally, we used STAR and CIRCexplorer2 to detect circular sisRNAs in the publicly available ENCODE tissue data (*n* = 113) (41); fractionated cell line data from K562 (*n* = 7) and HepG2 (*n* = 4) (41); immortalized bladder cancer cell lines (FL3, HCV29, T24; *n* = 6) (26); and a small RNase R treated HS68 cell line data set (*n* = 4) (42). The fractionated K562 and HepG2 data were generated by the labs of Brenton Graveley, UConn; Thomas Gingeras, CSHL; and Éric Lécuyer, IRCM (Supplementary File S1-2). In all instances, we disregarded circular sisRNA calls from the mitochondrial genome, non-coding RNA genes, and a length not within 30–10 000 base pairs (bp). Furthermore, we annotated the host intron of a circular sisRNA as the shortest intron fully containing the circular sisRNA. Circular sisRNA cases were visualized using IGV (43).

### Extracting unique intron annotations

We used the R packages “GenomicFeatures” and “GenomicRanges” (44) to extract genomic positions for all introns based on the GENCODE (v. 33) gene annotations (45). Isoforms were disregarded by collapsing overlapping intron annotations. To avoid exonic influence on read-depth and conservation, we excluded intronic positions overlapping exons from an alternative isoform. The introns have a large length variability, ranging from 1 bp to 721 292 bp (median = 1461 bp, *n* = 183 203, Supplementary Figure S1). To reduce the presence of artifacts and non-canonical introns, we only considered introns with a length between 100 and 10 000 bp (*n* = 153 582). Finally, we annotated the predicted circular sisRNAs with our resulting intron annotations based on genomic range overlap and close proximity of their 5’ coordinates (within 10 bp).

When relevant, we used BEDtools (46) to extract stranded sequences from the reference genome and SAMtools (47) to filter reads.

### Generating intron and gene expression matrices

We generated intron and gene expression matrices for all data sets mentioned above using HTseq count (48) in stranded mode and with loose read and intron overlap restrictions. Thus, pre-mRNA reads partially overlapping an intron were also counted. The relative intron expression is defined as (intron FPKM value)/(parent mRNA FPKM value), with a pseudocount of 1e-06 added to both numerator and denominator. For the ENCODE tissue data, we defined the tissue-specific expression as the average expression for all samples within each tissue.

### RNase R enrichment of circular sisRNAs

The total RNA-Seq immortalized human fibroblast (HS68) cell data, produced by Jeck *et al.*, consists of two replicates of RNase R treated and control samples. RNase R enzymatically degrades linear RNAs causing an enrichment in circular RNA species in the treated samples compared to the controls. We used the CIRCexplorer2 generated output to determine the number of circular-junction reads, with a threshold of at least two junction-spanning reads imposed. We counted the number of linear-splice reads across each intron using HTseq count after filtering reads with SAMtools. For each intron, we normalized the number of circular and linear junction reads as reads per million (RPM). Only circular junctions present in both the treated and control sample were included, and only linear junctions having a circular counterpart were included. We calculated the level of enrichment as the expression ratio of junctions in the RNAse R treated sample against its corresponding control sample. Enrichment was then defined as a ratio larger than one.

### Validation of select circular sisRNAs

We chose a small set of circular sisRNAs for validation based on their expression level in the NMIBC samples and on their detection in total RNA-Seq data from the cell line used (35).

We reverse transcribed 1 ug RNA from J82 and fractionated HepG2 cells in a 20 ul reaction with or without 1 ul MLV-RT enzyme (Thermo Fisher) but otherwise adhering to manufacturer's protocol. Then, we PCR amplified 2% with taq DNA polymerase (Thermo Fisher) for 35 cycles using primers listed in Supplementary Table S1. PCR fragments were visualized on a 1% agarose gel with 1X SYBR Safe (Invitrogen).

### Statistical analyses

We performed all statistical analyses in R (49,50) and generated plots using “ggplot2” (51). As a measure of central tendency we preferred medians for robustness to outliers and distributional asymmetry. In addition, we quantified variation using the standard deviation, and when relevant the interquartile range (IQR), which is explicitly stated when used. We used the two-sided non-parametric Wilcoxon rank-sum test to evaluate differential distributions. For comparing expression levels in BC risk classes, we required circular sisRNAs to have more than ten junction reads across ten samples. We also performed Fisher's exact test to evaluate the association between junction type (circular or linear) and RNase R enrichment for our discrete count data and used odds-ratio to quantify the association strength. Spearman's rank correlation coefficients were used to assess the relationships between pairs of stochastic variables. We conducted Kaplan–Meier survival analysis using a log-rank test with plots from the R packages “Survival” (52) and “Survminer”. In instances of multiple testing, Benjamini-Hochberg (BH) was applied to control the false discovery rate (FDR) with *q* < 0.1 considered significant. In addition, we report the fold change (FC) as a measure of the central difference between two distributions of interest and define it as the ratio between their means (a1 and a2), with a pseudocount added:


\begin{equation*}log \; FC = log_2\left(\frac{a1+1e-06}{a2+1e-06}\right)\end{equation*}

### Branch point annotations and related features

To annotate BP positions within all intron sets, we analyzed a comprehensive set of experimentally mapped and statistically inferred BP positions (here named Taggart BPs or simply BPs), which spans 16.8% of all human introns (14). We lifted their genomic coordinates to hg38 using UCSC’s web tool “Lift Genome Annotations” (53) and assigned them to our intron annotations (Supplementary Methods).

We defined the initial 3’ tail length as the distance between the branch point and 3’ splice site. All intronic lariats with negative 3’ tail lengths or lengths ≤1 bp, which may result from the truncation of non-exon overlapping intron annotations, were disregarded.

### Sequence context

We extracted sequences located from the 5’SS to 20 bp downstream of the 5’SS; 20 bp upstream of the 3’ SS to the 3’SS; and spanning 10bp on either side of the BP for all introns. For the BP sequences, all positions within 10 bp of the 3’ SS position were excluded from the sequence analyses to avoid effects on the conservation levels, etc. As a consequence, all introns with a BP to 3’ SS distance <10 bp were disregarded. We computed nucleotide frequencies using the “Biostrings” R package (54).

Bits were used as a measure of the information content at each position in the sequences, calculated using Kulback-Leibler divergence, and visualized as logo plots using the “ggseqlogo” R package (55) (Supplementary Methods).

### De novo motif discovery and analysis

We used STREME called by the XSTREME tool (with default settings), for comprehensive motif discovery (56,57). STREME (Sensitive, thorough, rapid enriched motif elicitation) is geared for large sequence sets (*n* > 50) and performs ungapped motif discovery (58,59). Motif significance is given by *E*-values, which measure the expected number of motifs with as high an enrichment in a random sequence set of the same size (*n* = 458).

As positive input, we used 101 bp long sequences centered at the branch points of stable lariats (based on CIRCexplorer2 positions). To remove potential splice site motifs, we trimmed ten bases at the splice site ends of the stable lariat host introns. As negative control input, we used similarly defined sequences centered at Taggart BPs from non-host introns from expressed genes (*n* = 30 836).

We used FIMO (Find individual motif occurrence) (60) for locating exact motif occurrences in the stable lariat BP sequence set and applied TomTom (61) to compare and align results from STREME with known target motifs of RNA binding proteins (RBPs) and micro-RNAs (62,63). To evaluate motifs depleted in branch point loci of stable lariats, we ran XSTREME with the background Taggart BP loci as the positive set and the BP loci of stable lariats as the negative set. Finally, a simple permutation scheme of the pooled sequences was used to generate ten randomly shuffled positive and negative sets used as input for XSTREME. All motifs were visualized by XSTREME using standard logo plots with bit sizes based on the Shannon entropy.

### Intron read-depth

We found the position-wise read-depth for the introns using mapped reads from all the NMIBC samples (*n* = 457). First, we only considered reads mapping directly to intronic loci and removed all linearly-spliced reads mapping across introns using their CIGAR strings. Subsequent, we selected reads mapping at least partially to the non-overlapping intron annotations defined above. Second, we summarized the read-depth for each sample using DeepTools's bamCoverage (64) and then summed the read-depth across all samples using UCSC’s application bigWigMerge (65) and their tool bedGraphToBigWig was used for file conversion as well as the tool “bwtools extract” (66). We averaged the position-wise read-depths per sample (Supplementary Methods).

A similar approach was used to obtain the read-depth of all non-filtered reads from the NMIBC samples.

### Intron sequence conservation

We evaluated the intronic sequence conservation using positional phyloP scores (67) from the 100-way vertebrate alignments downloaded from the UCSC Genome Browser (http://hgdownload.soe.ucsc.edu). The intronic position-wise phyloP scores were extracted using “bwtool extract”. We defined the whole intron conservation as the mean position-wise conservation across the entire intron region, with 10 bp at splice site ends removed (Supplementary Methods).

## RESULTS

### Identification and quantification of circular sisRNAs

To quantify the expression of circular sisRNAs in bladder cancer, we used the CIRCexplorer2 pipeline (37) to identify reads that traverse the splicing junction (Figure 1A) in total RNA-Seq data from a large cohort of patients (*n* = 457; UROMOL) with NMIBC (35). We initially identified and characterized 11 420 circular junctions supported by at least one junction-spanning read, which we later used to define a restricted set of circular sisRNAs. For data set overviews and data processing see Supplementary Figure S2. Overall, we observed a small fraction of junction-spanning reads, ranging from barely detectable to 7.7 reads per million (RPM) (median = 1.4 RPM), with a large variation between samples (Figure 1B). While most predicted junctions are supported by few junction-spanning reads (96% by less than five reads in total; Supplementary Figure S3A), some are highly supported, e.g., junctions derived from introns of *HNRNPK* (155 reads across 91 samples) and *WDR13* (135 reads across 93 samples) (Supplementary Figure S3B).

The RT often mis-incorporates nucleotides while traversing the 2’-5’ linkage, leaving uncertainty about the specific branch point location (11,14,68,69). Consistent with these studies, we found that many predicted junctions (31%) are annotated with the same 5’ splice site but differ in their branch point annotations (Figure 1C), usually only by a few bases (Supplementary Figure S3C). In the most extreme case, 49 predicted junctions had a shared 5’ splice site but mapped to distinct branch points. On the other hand, few junctions (0.4%) shared the same branch point while differing in their 5’ splice sites (Supplementary Figure S3D).

Predicted junctions from the same intron with the same 5’ splice site but differing BP coordinates likely represent the same potential circular RNA due to RT errors. They were therefore collapsed and represented by the junction with the highest number of supporting reads (Figure 1D, Supplementary Figure S2). Based on these collapsed junctions, we hereby identified 9216 junction clusters. We use the term cluster as some branch points differ widely in position and potentially represent alternative BP usage (Supplementary Figure S3C). One example is sisHNRNPK with BP positions that differ with up to 254 bp (Supplementary Figure S4A). However, as these potential sisRNAs are derived from overlapping loci, they have a high sequence similarity and are likely derived through a similar splicing event.

We compared the junction clusters detected by CIRCexplorer2 with ones detected by circRNA_Finder, DCC, and CIRIquant. We found that approx. 90% of potential circular sisRNA clusters overlap with at least one other detection method (Supplementary Figure S3E). Prominent circular sisRNAs detected by multiple methods include sisHNRNPK, sisWDR13, sisCNOT6, sisSLK and sisMBNL1 (Supplementary Table S2). In contrast to the other methods, CIRCexplorer2 is the only method, which accounts for intronic lariat biogenesis, and we found it had the highest agreement with intronic splicing and known splice sites. CIRCexplorer2 is designed to handle the intronic junction-reads through a re-alignment step (37).

Finally, the circular sisRNA (cluster) is defined as being derived from protein-coding genes, between 30 and 10 000 bp long, and expressed in at least two samples (*n* = 1827) (Supplementary Figure S2, Supplementary Tables S2–S4). Of these, the majority were categorized as stable lariats (55%) and the rest as intronic circles (45%) that span the entire intron (Figure 1A, E). In concordance with stable lariats containing a 2’-5’ linkage and intronic circles proposed as having a 3’-5’ circularizing junction, stable lariats have more uncertainty at their 3’ end (mean of 2.38 predicted junctions) than intronic circles (mean of 1.68 predicted junctions) (Figure 1F). The 3’ end of intronic circles are not expected to represent classic BPs but rather the 3’ SS linked to the 5’ SS of the whole-intron RNAs.

The highest expressed circular sisRNA, sisHNRNPK (chr9:83 975 758–83 976 994; hg38; Supplementary Figure S4A), first discovered here to our knowledge, has multiple predicted junctions (*n* = 27) with differing 3’ ends, and is supported by the highest number of junction-spanning reads (*n* = 570) across the highest number of samples (*n* = 222) (Figure 1F, G, Supplementary Table S2). In contrast, the highest expressed intronic circle, originating from *WDR13* (sisWDR13; chrX:48 598 958–48 599 352, Supplementary Figure S4B), has two predicted junctions (differing by one nucleotide, providing less ambiguity about the 3’ end), and is supported by the second highest number of junction-spanning reads (*n* = 136) across almost a hundred samples (*n* = 94) (Figure 1F, G, Supplementary Table S2).

### Validation of circular-junctions and select circular sisRNAs

To increase confidence in the validity of circular junctions detected by CIRCexplorer2, we investigated the enrichment of circular junction-spanning reads upon RNase R treatment, which enzymatically degrades linear RNAs (42). We only included circular junctions detected in both treated and untreated samples. Encouragingly, the majority (83.7–84.1%) of intronic circular junctions in human fibroblast (HS68) cell line data (42) were enriched upon RNase R treatment, which is more than the corresponding linear splice-junctions (25.3–29.5%) (Figure 1H). The association between enrichment and junction type (circular or linear) were statistically significant (*P*-values < 2.2e–16; odds-ratio of 15.4 (replicate 1) and 12.2 (replicate 2); Fisher's exact test).

To confirm the authenticity of individual circular sisRNAs, we attempted to experimentally validate several of our findings (sisHNRNPK, sisWDR13, sisCNOT6 and sisARHGEF10L) in the human bladder cancer cell line J28 and human liver cancer cell line HepG2 using RT-PCR and Sanger sequencing (Figure 1I, Supplementary Figure S5). Junction-spanning primers are shown in Supplementary Table S1. Both sisHNRNPK and sisCNOT were successfully detected by both methods, while the intronic circle sisARHGEF10L was detected using RT-PCR (Figure 1I). Although we could not detect sisWDR13 in our cell lines, this molecule has previously been validated in other data (14) (Figure 1I). In addition, investigating total RNA-Seq J28 data showed little support for sisWDR13 expression in the cell line, which may explain the unsuccessful validation.

### Circular sisRNAs and their host introns have distinct features

**Figure 2.**
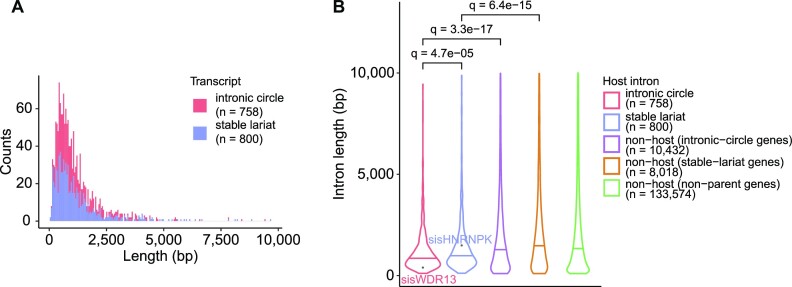
Circular sisRNA and host intron lengths. (**A**) Length distributions of intronic circles (red) and stable lariats (blue). bp = base pair. (**B**) Intron length distributions (violin plots) with medians indicated (bars). *q* < 0.1 for all comparisons. *q*-values are obtained through Benjamini-Hochberg control of the false discovery rate (FDR) during multiple testing (Wilcoxon rank-sum tests). Host introns are defined as the unique non-overlapping intron set.

**Figure 3.**
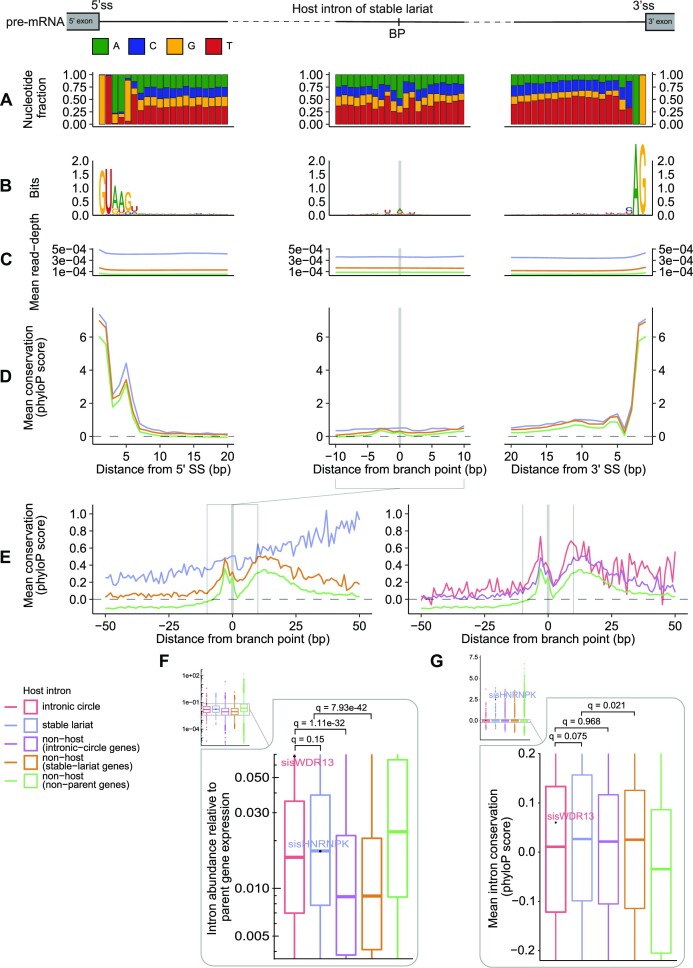
Position-wise characterization of stable-lariat intron sequences. (A–E) Position-wise summaries based on sequence alignment at the 5’ SS (column one), BP (column two) and 3’ SS (column three). When considering the branch point region, ten nts are removed from the intron ends to remove the splice site signal. Group sizes for column one and three are the same as in Figure 2, while column two depends on Taggart *et al.* BP annotations within introns; intronic circles (*n* = 314), stable lariats (*n* = 394), non-host introns (intronic-circle host-genes; *n* = 2818), non-host introns (stable-lariat host-genes; *n* = 3272), and non-host introns (non-host genes; *n* = 24 746). (**A**) Positional nucleotide distributions of introns hosting stable lariats. A = adenine, U = uracil, G = guanine, C = cytosine. (**B**) Logo plots depicting information content of the nucleotide distributions. (C–G) Group colors as shown in the bottom left corner of the figure. (**C**) Mean position-wise read-depth found across all NMIBC samples. (**D**) Mean positional conservation scores (phyloP100Way score). (**E**) Mean positional conservation scores (phyloP100Way score) for the 100 bases adjacent to the BP. Group colors indicate lariat class containing the BP used for sequence alignment. The plot is split in two for stable lariats and intronic circles. (**F**) Distribution of the log-scaled intron to parent gene expression ratios (FPKM ratios) with the three quartiles highlighted. Same intron classes as in Figure 2. (**G**) The mean intron conservation (phyloP100Way score) distributions with the three quartiles enlarged. Same intron classes as in Figure 2.

Several features have been suggested to promote stability of circular sisRNAs, including length, 3’ tail size, branch point nucleotide (nt), sequence context and enriched branch point motifs (11,12,18–21,24). We investigated the presence of these and other features across our set of circular sisRNAs and their host introns (Figures 2 and 3, Supplementary Figures S6–S9). To avoid bias, we based the analyses on a set of non-exon-overlapping introns found to be expressed in our data and disregarded alternative isoforms (*n* = 153 582). We defined a set of high-confidence circular sisRNAs with 5’ splice sites concordant with these (*n* = 1558; Supplementary Figure S2).

We found that the length of intronic circles (median = 854 bp) and stable lariats (median = 982 bp) in bladder cancer are larger than previously reported for the cytoplasm of healthy tissues in vertebrates (majority between 100 and 500 bp) (12), in porcine tissue (>95% of intronic circRNAs had a length < 600 bp) (15), and in cell line data (19) (Figure 2A). For both sisRNA classes, we compared the lengths of circular sisRNA host introns to the lengths of conventional unstable introns from the same genes (referred to as same-gene non-host introns) as well as to the larger class of introns from non-parent genes. The same-gene non-host introns were used as a control with the same expression properties as circular sisRNA host introns, while the full set of introns from non-parent genes is a heterogeneous set with high expression variability. In all instances, the circular sisRNA host introns were shorter than non-host introns (*q*-values < 6.4e-15, log fold change (FC) < –0.61; Wilcoxon rank-sum test; due to multiple testing, we obtained *q*-values by Benjamini–Hochberg control of the false discovery rate (BH FDR controlled); Figure 2B, Supplementary Figure S1 and S2).

Short 3’ SS to BP distances promote stability for sisRNA in yeast (24) while stable lariats have been shown to be associated with lack of a 3’ tail in other organisms (11,19,42,70). In our analysis, however, the inferred 3’ tail length (BP to 3’ SS distance; median = 33 bp) of stable lariats is similar to conventional lariats (median = 27 bp), with intronic-circle host-introns having the shortest tail lengths (median = 24 bp; Supplementary Figure S6A). The BP nucleotide frequencies also showed some change between stable lariats (48% canonical adenine (13,14,34,69)) compared to same-gene non-host introns (66% adenine) (Supplementary Figure S6B). We used the previously annotated and comprehensive BP map generated by Taggart *et al.* (13,14) to assign BP positions for all our intron subsets, which is subsequently used for all branch point analyses (Supplementary Figure S2A). Comparing the Taggart BPs with the CIRCexplorer2 BPs (only possible for stable lariats), we found a median distance of 0 bp (mean = 7.7 bp) and ∼52% of BPs had a distance <20 bp (Supplementary Figure S6C). While the exact BP position is hard to recover due to low read-coverage and the nature of the 2’-5’ junction, the adjacent BP region showed similar features using either CIRCexplorer2 or Taggart BP annotations (Supplementary Figure S6D).

Turning to the larger sequence context of BPs, we discovered that the degenerate BP consensus motif is diminished in the host introns of stable lariats but strongly present for intronic circle host-introns and non-host intron classes (Figure 3A, Supplementary Figure S7 and S8A). Compared to other introns, the host introns of stable lariats have a high uracil (U) frequency adjacent to the branch point position (Figure 3A, Supplementary Figure S7 and S8A). Lastly, the highly-conserved sequence motifs of both canonical splice sites are observed in all intron classes (Figure 3A, B, Supplementary Figure S7 and S8A, B).

**Figure 4.**
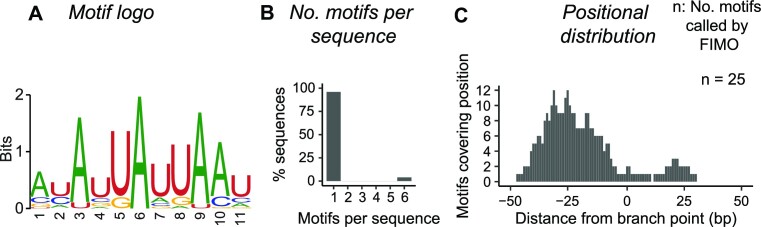
Significantly enriched motif, ‘AYAUUAUUAAU’, in the branch point locus of stable lariats. (A–C) A 100 bp region adjacent to the BP position is considered when performing de novo motif discovery analysis. Nucleotide and subset abbreviations: A (adenine), U (uracil), G (guanine), C (cytosine), Y = {C, U}. (**A**) Sequence logo of the ungapped motif with highest enrichment in stable lariats. (**B**) Number of motif occurrences per sequence. Motif occurrence is based on detection by FIMO (Find motif occurrence). (**C**) Motif distribution across the branch point locus based on the FIMO output.

We further performed de novo motif discovery of enriched sequence motifs in the vicinity of stable lariat BPs using STREME (56,59), which revealed two significant motifs: ‘AYAUUAUUAAU’ and ‘UUUAAAA’ (*E*-value < 0.05, Figure 4, Supplementary Figure S9A, B). Ten reruns on permutated data did not yield any motifs of similar significance. The AU-rich and eleven nucleotide long ‘AYAUUAUUAAU’ motif is found in 43 (9.4%) introns hosting stable lariats and is 6.2x more prevalent than in non-host introns. Interestingly, the motif resembles a repetition of the degenerative core part of the human BP consensus motif, 5’-UNA-3’, and may promote alternative BP usage (71). The ‘AYAUUAUUAAU’ motif is most commonly found upstream of the BP position (median position = –24 bp from the BP) and once per sequence (96%) (Figure 4). The two enriched motifs do not match known RBP binding sites or miRNA target sites. Finally, we also considered significant motif underrepresentation, which led to the finding of two depleted GC-rich motifs (Supplementary Figure S9C, D). For all four motifs, no differential expression is observed between stable lariats with and without motif occurrence (Supplementary Figure S9E). This could be caused by the high sparsity of the junction-read data or as a consequence of the motifs not relating to stability but potentially function.

Lastly, we investigated if the entire host introns of circular sisRNAs were highly expressed compared to non-host introns (Figure 3F). In general, the expression of circular sisRNA junctions correlated positively with whole-intron expression across samples, with the circular junctions supported by many reads showing the strongest correlation (Supplementary Figure S10). Additionally, the average read-depth for all intron classes is evenly distributed across the entire intron length, with a slight increase toward the ends (expected to be caused by pre-mRNA reads; Figure 3C, Supplementary Figure S8C). For both circular sisRNA classes, the relative host-intron expression is significantly higher than for the corresponding same-gene non-host-introns (*q*-values < 1.2e-32; log FC > 0.82; Wilcoxon rank-sum test; BH FDR controlled, Figure 3F). Thus, a circular sisRNA expression signal can be observed at the whole-intron level.

In total, two circular sisRNA host introns have a relative expression larger than one. They host sisPABPN1 (chr14:23 323 484–23 323 964, ratio = 3.82) and sisRCC1 (chr1:28 508 161–2 8508 618, ratio = 1.02). sisPABPN1 is an intronic circle derived from an intron occasionally retained in the mature parent mRNA. sisRCC1 is a stable lariat derived from an intron also giving rise to SNORA73B, which can partially explain the large intron expression signal.

### Introns hosting stable lariats have conserved sequences across vertebrate species

High cross-species sequence conservation for circular sisRNA host introns may indicate functional importance. In addition, position-wise conservation can help pinpoint functionally important loci. Not surprisingly, introns tend to have a low mean position-wise conservation (∼40% of all mean intron phyloP-scores are within 0.1 of zero, Figure 3G). Interestingly, introns hosting stable lariats were found to have a significantly higher intron conservation compared to same-gene non-host introns (*q*-value = 0.021, log FC = 2.58; Wilcoxon rank-sum test, BH FDR controlled).

Most notably, the gene *MBNL1* hosts two highly abundant and highly conserved circular sisRNAs (chr3:152 455 578–152 456 060 and chr3:152 445 540–152 446 580; mean whole-intron conservation scores of more than two). The *MBNL1* gene encodes an RNA-binding protein, which regulates alternative mRNA splicing, and is known to be deregulated during myotonic dystrophy (72,73). Two other highly conserved cases are derived from two neighboring introns in *HNRNPK* (chr9:83 974 619–83 975 461 and sisHNRNPK; Supplementary Figure S4A). The high sequence conservation is a product of a several hundred bp long and highly-conserved region overlapping the two introns.

Further, we examined the conservation levels of intronic loci important for proper splicing (Figure 3D, E, Supplementary Figure S8D). As expected for introns in general, the consensus motifs of both splice sites are highly conserved and the degenerative branch point motif of non-host introns have an elevated conservation (Figure 3D, E, Supplementary Figure S8D). Host introns of circular sisRNAs have a larger position-wise mean conservation than non-host introns at almost all positions at the SS and BP loci, with the largest differences being observed at the BP region (71.3% of positions adjacent to the BP have log FC scores > 1 for stable lariats, Figure 3E). Interestingly, the mean phyloP-score distribution across the BP loci of intronic-circle host-introns resemble that of non-host introns rather than that of the stable lariat host-introns (only 35.6% of positions adjacent to the BP have log FC scores > 1 for intronic circles, Figure 3E). The high conservation levels at the branch point region of introns hosting stable lariats may indicate that this region is of particular importance for their stability or function.

### Circular sisRNAs have tissue-specific expression patterns

**Figure 5.**
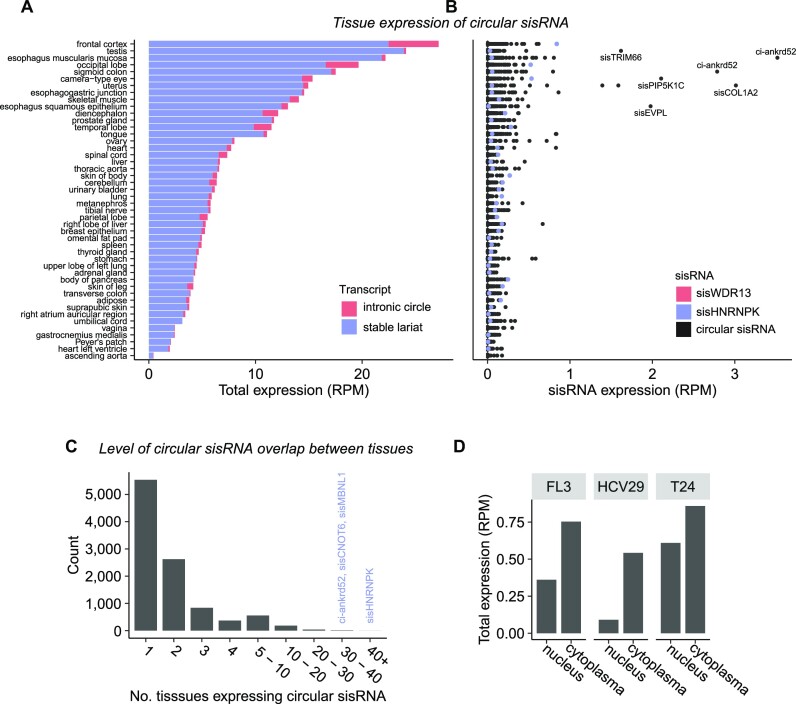
Circular sisRNA distribution across cell fractions and tissue types. (**A**) Total expression of circular sisRNAs within each tissue. Colors indicate type of transcript. RPM = reads per million. (**B**) Expression levels for individual circular sisRNAs within each tissue. Boxplots with outliers shown. Colors mark specific sisRNAs, with sisWDR13 being absent in all tissues. (**C**) Counts of how many tissue types (n = 46) each circular sisRNAs is expressed in. (**D**) Total circular sisRNA expression in nuclear and cytoplasmic fractions of three bladder cancer cell lines.

To investigate tissue-specific expression patterns of circular sisRNAs, we utilized the CIRCexplorer2 pipeline on 113 total RNA-Seq adult and fetal samples from 46 tissues from the ENCODE project (41). By this approach, a large set of circular sisRNAs (*n* = 10 162) supported by at least two junction-spanning reads were profiled across all tissues (Supplementary Table S5, Supplementary Figure S2B). The circular sisRNA set detected in NMIBC tumors share limited overlap with the combined tissue set (*n* = 215) and with the urinary bladder tissue in particular (*n* = 34). The total expression level (ranging from 0.43 to 27.18 RPM) and the number of circular sisRNAs (ranging from 5 to 1690) are found to be highly tissue specific (Figure 5A, B, Supplementary Table S6). The total expression is usually driven by few highly expressed circular sisRNAs, with the top 10% of circular sisRNAs contributing between 27% and 75% of the total tissue expression (Supplementary Figure S11A). In addition, the majority (54%) of circular sisRNAs are expressed in only one tissue type (Figure 5C).

This generalizes the findings made by Zhang *et al.* (19), where some ciRNAs were found to be broadly expressed (ci-ankrd52), while others had a highly tissue and cell-type specific expression pattern in humans. Robic et al. also showed that the number of intronic circRNAs differed between tissues in bovine and porcine data (74). Here, we also detected 238 circular sisRNAs in at least ten different tissues, including sisHNRNPK expressed in 44 tissues as well as sisMBNL1 and ci-ankrd52 present in 38 different tissue types (Figure 5A, B).

In general, all tissue types have a low fraction of intronic circles compared to stable lariats, with an intronic circle fraction of at most 20% (mean = 7.2%; Figure 5A, Supplementary Table S6). The urinary bladder tissue has an intronic circle percentage of 5.7% (34 of 599 circular sisRNAs), which is roughly ten times lower than the intronic circle percentage for the NMIBC samples (Figure 5A, Figure 1E). Interestingly, tissue samples from the central nervous system (CNS) tend to have the highest intronic circle fractions, with all CNS tissues having a fraction larger than 10% (Supplementary Table S6). The accumulation of intronic circles in the CNS is concordant with previous findings of circRNAs, including intronic circles in mice, tending to accumulate in slow-dividing cells and aging brain tissue across multiple species (16,39,75–77). CNS studies could thus be particularly well-suited for yielding insights into the biological functions of intronic circles.

### Circular sisRNA are found in multiple cell fractions

Intronic lariats are produced during transcription and are typically thereafter rapidly degraded by the debranching enzyme (Dbr1) and exonucleases, a process taking place in the cell nucleus. Exportation of circular sisRNAs to the cytoplasm can promote stability due to the physical separation of the circular RNA containing the 2’-5’ junction and Dbr1 (11,12). We investigated circular sisRNA presence in the cytoplasm of fractionated cell lines originating from bladder cancer (T24 and HCV29) and T24 derived lung metastasis (FL3), using previously generated local total RNA-Seq data (26). A small set of circular sisRNAs (n = 149) was detected across the nuclear and cytoplasmic fractions of the three cell lines (Supplementary Table S7A). Intriguingly, circular sisRNAs are present in both the nuclear (T24 = 49, HCV29 = 2, FL3 = 8) and cytoplasmic (T24 = 70, HCV29 = 6, FL3 = 20) compartments, with little overlap in circular sisRNAs between the two fractions (overlap for T24 = 3, HCV29 = 0, FL3 = 0). For all three cell lines, the cytoplasmic fraction had the highest total circular sisRNA expression (Figure 5D, Supplementary Table S7A).

Similar results were observed for the ENCODE cell lines K562 (*n* = 11; 5630 circular sisRNAs identified) and HepG2 (*n* = 4; 880 circular sisRNAs identified) (41) (Supplementary Figure S11B, C). For both K562 and HepG2, circular sisRNAs found in the cytosol have the lowest median lengths of 399 bp (K562) and 538 bp (HepG2) as well as the lowest corresponding interquartile range (IQR) (Supplementary Table S7B, C). This indicates that smaller intronic molecules tend to be exported to the cytoplasm more frequently or that they possess the highest stability in the cytosol. Finally, similar proportions of intronic circles were found across all cell fractions (within each cell line), with the BC cell lines having the highest percentage (Supplementary Table S7).

### sisHNRNPK and sisWDR13 expressions are correlated with clinical outcomes

**Figure 6.**
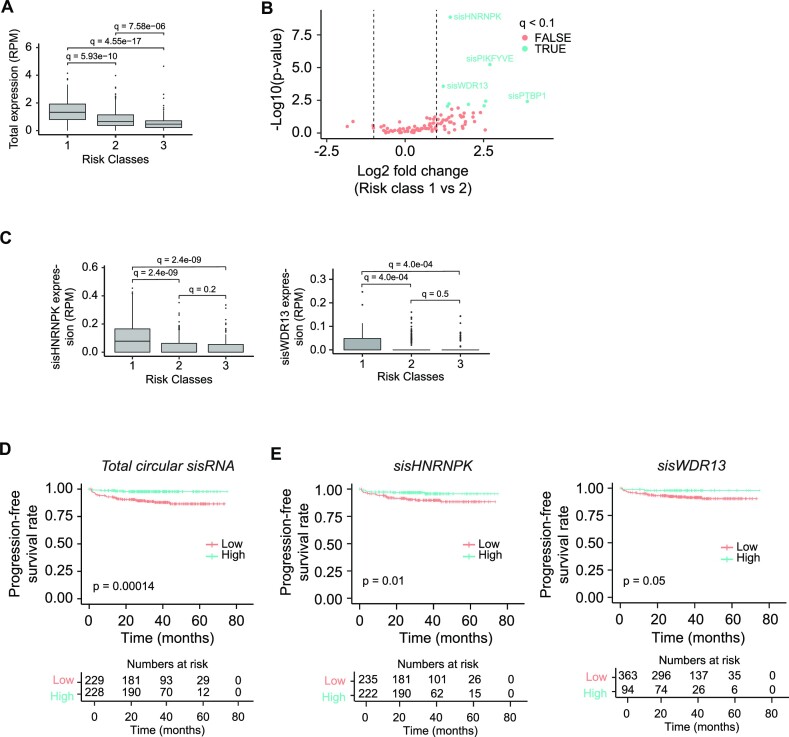
sisHNRNPK and sisWDR13 expressions are correlated with clinical outcomes. (**A**) Total expression of circular sisRNAs in NMIBC tumors divided into prognostic risk classes. *q*-values are obtained from Wilcoxon rank-sum tests with FDR control using the Benjamini–Hochberg (BH) method. RPM = reads per million. (**B**) Differential expression analysis of circular sisRNAs between risk class 1 (*n* = 96) and 2 (*n* = 232) tumors from patients with NMIBC. The log_2_ fold changes (class 1 versus class 2) are plotted against the negative log_10_(*P*-values). Colors indicate if circular sisRNAs are significantly (blue) or not significantly (red) differentially expressed after BH FDR control (*q* < 0.1). Vertical lines indicate a log FC > 1 or < –1. (**C**) Expression of sisHNRNPK (left) and sisWDR13 (right) in risk class 1, 2 and 3 tumors. *q*-values obtained as in panel A. (**D**) Kaplan–Meier progression-free survival plots for the total circular sisRNA expression in the NMIBC samples (*n* = 1827). Median expression cut-off of 0.67 RPM is used. *P*-values are obtained by Log-Rank Test. RPM = reads per million. (**E**) Survival plots for sisHNRNPK (left) and sisWDR13 (right) in patients with NMIBC. Median expression used as cut-off: sisHNRNPK = 0 RPM and sisWDR13 = 0 RPM. *P*-values are obtained by Log-Rank Test.

To assess the potential clinical importance of circular sisRNAs, we evaluated their expression in three major risk classes (RCs) of NMIBC with distinct molecular characteristics and clinical outcomes (class 1: low-risk tumors with mainly good prognosis, *n* = 96; class 2: high-risk tumors characterized by poor prognosis, *n* = 232; and class 3 tumors: low/intermediate risk tumors, *n* = 129) (35). Class 1 tumors showed the highest total circular sisRNA expression (median = 1.32 RPM), while class 2 (median = 0.65 RPM) and class 3 tumors (median = 0.47 RPM) had significantly lower expression levels (*q* < 6e-10; log FC > 0.75; Wilcoxon Rank-Sum Test; BH FDR controlled, Figure 6A). By conducting pairwise differential expression analyses between the three risk classes for the highly abundant circular sisRNAs (*n* = 97), we found that 9 cases were differentially expressed between class 1 and 2 tumors, and 16 between class 1 and 3 tumors (*q* < 0.1; Wilcoxon Rank-Sum Test; BH FDR controlled, Figure 6B, Supplementary Figure S12A). No circular sisRNAs were differentially expressed between risk class 2 and 3.

The two most abundant circular sisRNAs, sisHNRNPK and sisWDR13, showed the most perturbed expression profiles between risk class 1 and 2, and 1 and 3 tumors (Figure 6B, Supplementary Figure S12A). Both sisRNAs were significantly higher expressed in class 1 tumors than in class 2 and 3 tumors (*q* < 5e-04; log FC > 1.2; Wilcoxon Rank-Sum Test; BH FDR controlled, Figure 6C). In addition, all pairwise comparisons between risk classes of both host intron and parent gene expression were significant (*q* < 0.1 for all comparisons; Wilcoxon Rank-Sum Test; BH FDR controlled, Supplementary Figure S12B, C), except for the sisHNRNPK-intron between class 1 and 2 (*q* = 0.2, Wilcoxon Rank-Sum Test; BH FDR controlled, Supplementary Figure S12B).

Risk class 2 has recently been shown to be further subdivided into subclasses 2a (highest progression rate) and 2b (higher immune cell infiltration and lower progression rate) (78). Curiously, the total circular sisRNA expression was lowest in risk class 2a, including the individual expression of sisHNRNPK and sisWDR13 (Supplementary Figure S13). Here, RC 1 showed the highest circular sisRNA expression, RC 2b and 3 showed an intermediate expression, while RC 2a had the lowest overall circular sisRNA expression (Supplementary Figure S13). The initial higher circular sisRNA expression in RC 2 compared to RC 3 (Figure 6A), may thus be driven by the subgroup 2b with an intermediate prognosis.

Based on the observations of circular sisRNA deregulation in subclasses of NMIBC with distinct biological characteristics and prognosis, we evaluated the prognostic potential of circular sisRNAs, particularly, sisHNRNPK and sisWDR13. Interestingly, we found that progression-free survival correlated positively with the total circular sisRNA expression (*P* = 1.4e-04) as well as the expression of both sisHNRNPK (*P* = 0.01) and sisWDR13 (*P* = 0.05) (Log-Rank Test, Figure 6D, E). While the sisWDR13 parent gene and its host intron yielded similarly significant results (*P* < 0.01, Log-Rank Test, Supplementary Figure S14), the expression of the *HNRNPK* gene or the sisHNRNPK-intron were not able to distinguish patients with good prognosis from patients with poor prognosis (*P* > 0.34, Log-Rank Test, Supplementary Figure S14). Looking at other prominent circular sisRNAs, we found no significant correlation with progression-free survival. This could either be because the sisRNA expression could not distinguish between patient groups with high and low progression-free survival rates (sisCNOT6, *P*-value = 0.97; sisFLOT1, *P*-values = 0.94) or potentially because we did not have the statistical power to detect differences caused by low sisRNA resolution and unbalanced group-sizes (sisARHGEF10L, *P*-value = 0.052; sisMBNL1, *P*-values = 0.22). sisSLK had a negative correlation with progression-free survival (*P* = 0.028), however, similar results were also found for the parent gene expression.

## DISCUSSION

In this study, we comprehensively characterized the overall landscape of circular sisRNAs in early stage bladder cancer. We identified two distinct classes of circular sisRNAs, i.e. stable lariats and intronic circles, which localize to both nuclear and cytoplasmic compartments and show tissue specific expression patterns. We found that circular sisRNA host introns are short introns, which contain canonical splice site motifs, and are higher expressed than other introns from the same gene. The stable lariats are derived from conserved introns with an ‘AU’-rich motif enriched upstream of the BP position, and an overall high uracil frequency at the BP region. In contrast, intronic circles are derived from lowly conserved introns with BP loci similar to conventional introns. In addition, we showed that circular sisRNAs are differentially expressed between prognostic bladder cancer risk classes and that the total circular sisRNA expression as well as individual cases correlate with progression-free survival.

We have shown that circular sisRNAs can be studied and novel cases identified through analysis of large sets of total RNA-Seq samples, despite low expression levels. Future total RNA-Seq data sets with higher read-depth and more samples, will improve the detection power and resolution, and enable further delineation of regulatory and functional hypotheses (79,80). During this study, we chose a relatively lenient read-support level for circular sisRNA junctions, which should be present in at least two samples. We found a sensitivity approach was needed to comprehensively detect rare circular junction reads, particularly, for the 2’-5’ traversing reads. Selecting circular sisRNAs with higher support may remove true positives with low expression performing local gene regulation, as suggested by Zhang *et al.* (19).

Large-scale human BP mapping efforts recently led to the discovery of 3'-5’ intronic circles, with 3% of all inferred BPs mapping to the 3’ SS position (14). Interestingly, we found that ∼45% of circular sisRNA in bladder cancer are intronic circles spanning the entire introns, which is a vastly larger fraction than in normal tissue. This could be caused by perturbations of intronic circle synthesis or transcript degradation in cancer, with similar deregulation being observed for other ncRNA species (27,81). Some circular sisRNA clusters contained junction-reads supporting both stable lariats and intronic circles (e.g., sisPAPOLA and sisCLK1; Supplementary Table S2), which may further support the hypothesis that intronic circles are derived from a lariat intermediates through a post-transcriptional event as suggested by Taggart et al. (13).

We characterized features suggested to reduce the lariat-debranching efficiency of Dbr1 and thus affect circular sisRNA stability (11,12,18,19,21,24). The exact mechanisms providing stability may differ between the sisRNA subclasses. For intronic circles, the expected 3’-5’ junctions are inaccessible by Dbr1 and thus inhibits regulation and degradation through the conventional lariat turnover pathway (82).

In contrast, stable lariats require other methods to escape debranching. Interaction between the lariat substrate and the active site of Dbr1 depends on the existence of a 3’ tail (82,83). In yeast, a small BP to 3’ SS distance has been shown to be sufficient in stabilizing some sisRNAs (24). We hypothesized that a similar mechanism may be present in humans due to the association between stable lariats and at least partial loss of the 3’ tail (5,11,19). A shorter initial 3’ tail length may imply a quicker degradation by exonucleases and an increased stability of the intronic lariat. However, no such trend could be observed in our data and the digestion of the 3’ tail may simply be a product of stable lariats persisting sufficiently long for nucleases to act on them.

The stable lariats had a slightly lower fraction of the canonical branch point nucleotide adenine (A) and a reduced canonical BP consensus motif (14,34,69). Cytosine (C) has previously been found to be a frequent BP nucleotide of stable lariats in multiple species including human, mouse, pigs and zebrafish cells and is known to reduce the debranching efficiency of Dbr1 (12,18–21). We also observed an increased usage of C at the BP of stable lariats, however, not to the degree previously reported. This may in part be due to the different data preparation procedures, including absence of RNase R treatment leading to a generally lower read-depth at the circularizing junction and a lower BP resolution.

The Taggart BPs annotated to stable lariats generally differed slightly from those derived with CIRCexplorer2. This may partly be caused by processing differences, where Taggart et al. used a U2snRNA:pre-mRNA base-pairing model to infer the true BP position in place of RT skipping (14). However, differing BP coordinates may also be a product of alternative BP usage across tissues (84), or between healthy and cancer tissue (85,86). Thus, a focus on the BP loci rather than the exact BP positions, is expected to produce the most robust results. RNase R treatment of samples in future studies may further help resolve the BP loci of known circular sisRNAs (11,12,40,42,70,79), which could give novel insights into how they obtain stability and potential disruption in cancer.

We found that circular sisRNAs and their host introns are relatively short, which might affect debranching by Dbr1 or increase their chance of being exported to the cytoplasm (11,12,19). Correspondingly, we detected circular sisRNAs in the cytoplasm of human cell lines, which were significantly shorter than in other cell fractions. Similarly, cytoplasmic circular sisRNAs have previously been detected in zebrafish, frog, chicken, mouse, and human cells, and were derived from short introns and suggested to have a regulatory role in mRNA translation (11,12). Our results thus further support that a subset of circular sisRNAs may perform their function in the cytoplasm.

Sequence conservation, which implies purifying selection to preserve function, was significantly higher for introns hosting stable lariats than same-gene non-host introns, with highly conserved cases including sisHNRNPK and sisMBNL1. This may be attributable to sequence elements important for the stability and function of circular RNAs. The position-wise mean conservation immediately upstream and downstream of the BP were several orders of magnitude higher than for the non-host intron classes. This suggests that conserved sequence motifs at the BP locus may play a vital role in intronic lariat stability or function. Interestingly, similar results were not found for the intronic circles, indicating that the elevated sequence conservation is unique for the stable lariats and their BP regions.

Circular sisRNAs may have potential as prognostic biomarkers in bladder cancer, similar to previous findings for exonic circular RNAs (circRNAs) (26,27). Our results show that the overall expression of circular sisRNAs differ between prognostic risk classes. Deregulation of circular sisRNAs may in principle function as a marker for cancer progression, as patients with low circular sisRNA expression have a poorer prognosis and the expression correlates with progression-free survival. In particular, the expression of sisHNRNPK positively correlates with progression-free survival, while the expression of the parent gene, *HNRNPK*, does not.

sisHNRNPK is highly conserved and has different characteristics compared to other stable lariats and thus appears to obtain stability through other means. *HNRNPK* is a well-described DNA/RNA binding protein involved in pre-mRNA processing, including RNA splicing (87). Furthermore, *HNRNPK* is involved in the tumorigenesis of multiple cancer types (88–92), with specific lncRNA association of hnRNP-K previously shown to either promote or inhibit self-renewal in bladder-cancer stem-like cells (88,93,94). *HNRNPK* expression has also been shown to be upregulated in bladder cancer and correlate with poor clinical outcome in patients (92). We hypothesize that sisHNRNPK may play an autoregulatory function to finetune the transcription level of its parent gene needed for normal cell function, as negative feedback mechanisms have evolved repeatedly for RNA binding proteins (95–100) and autoregulation has previously been described for ci-ankrd52 in HeLa cells and sisR-1 in *Drosophila Melanogaster* (19,23).

In order to produce a high-confidence circular sisRNA set, we filtered out circular sisRNAs originating from ncRNA genes (e.g., MALAT1) and having a mitochondrial origin. However, recent studies indicate that some of these circular junctions could have the potential to represent true circular sisRNAs and could be a point of further study (15,16).

In summary, we have profiled the expression of circular sisRNAs across healthy tissues and in bladder cancer, and evaluated their biological characteristics and clinical associations. Our study further identified individual circular sisRNAs that stand out in terms of expression levels, evolutionary conservation, or clinical correlations, including sisHNRNPK, sisWDR13, and sisMBNL1, which may act as candidates for the experimental functional characterisation of circular sisRNAs and their role in cancer.

## Supplementary Material

zcad041_Supplemental_Files

## Data Availability

The NMIBC RNA-Seq data was initially published by Hedegaard et al. and is available under controlled access. The access process can be initiated by contacting Lars Dyrskjøt (lars@clin.au.dk). As a consequence of privacy laws, data will only be available following new approvals by ethical committees and data protection agencies. The RNase R treated sequencing data can be found at the Short Read Archive (SRA050270). Branch point tables from hg19 introns are available as supplementary data for Taggart *et al.* (http://fairbrother.biomed.brown.edu/data/Lariat2016/). The total RNA-Seq tissue data are accessible through the ENCODE project (https://www.encodeproject.org/; see Supplementary File S3). Fractionated K562 and HepG2 cell line data are also available through ENCODE (see Supplementary File S1-2). Total RNA sequenced data from the bladder cancer cell lines and fractionated cell lines are deposited in NCBI’s Gene Expression Omnibus (GSE100971). All other relevant data and code are available from the corresponding author upon request, through the current release at Zenodo (https://doi.org/10.5281/zenodo.8147213), or the newest update at GitHub (https://github.com/JakobSkouPedersenLab/circular_sisRNA_analysis.git).
