# Supplementary material for: Circular stable intronic RNAs possess distinct biological features and are deregulated in bladder cancer
Source: NAR Cancer. 2023 Aug 7;5(3):zcad041. doi: 10.1093/narcan/zcad041 (PMC10405568; doi:10.1093/narcan/zcad041)
Supplement: zcad041_Supplemental_Files [file zcad041_supplemental_files.zip › Supplementary_material.pdf]

Circular stable intronic RNAs possess distinct biological features and are deregulated in bladder cancer

Asta M. Rasmussen[1,2,3,†], Trine Line H. Okholm[4,†], Michael Knudsen[1,2], Søren Vang[1,2], Lars Dyrskjøt[1,2], Thomas B. Hansen[5], and Jakob S. Pedersen[1,2,3,*]

[1]Department of Clinical Medicine, Aarhus University, Aarhus 8000, Denmark,
[2]Department of Molecular Medicine (MOMA), Aarhus University Hospital, Aarhus 8000, Denmark,
[3]Bioinformatics Research Center (BiRC), Aarhus University, Aarhus 8000, Denmark,
[4]Departments of Otolaryngology-Head and Neck Surgery and Microbiology & Immunology, University of California, San Francisco, CA, USA,
[5]Department of Molecular Biology and Genetics (MBG), Aarhus University, Aarhus 8000, Denmark

Supplementary Material

SUPPLEMENTARY METHODS **2**

SUPPLEMENTARY TABLE LEGENDS **3**

SUPPLEMENTARY FILE LEGENDS **4**

SUPPLEMENTARY FIGURES **5**

SUPPLEMENTARY METHODS

Branch point annotations and related features

For the branch point analyses, we did not define the stable lariat BPs as the last nucleotide position from the CIRCexplorer2 predicted stable lariat coordinates, due to low read coverage and high uncertainty of the exact BP position. Instead, we included a comprehensive set of mapped BP positions (named Taggart BPs), which spans 16.8% of all human introns (14). We lifted the Taggart BP positions from hg19 to hg38 using UCSC's web tool "Lift Genome Annotations" (53) and assigned the Taggart BPs to an overlapping intron from the non-overlapping intron annotations. BP assignments to individual introns were performed using overlap in their genomic ranges, by utilizing the "GenomicRanges" R package. We extracted the nucleotides for all BP positions using BEDtools (46). In addition, to account for the differences in BP mapping by CIRCexplorer2 and Taggart et al., we used two sets of BPs mapping within host introns of stable lariat: Predicted BP positions given by the CIRCexplorer2 output and BP positions predicted by Taggart et al. This, in order to compare similarities in results based on the two sets. Some variability was observed in the BP location between the two sets, however, the resulting BP locus tended to have similar nucleotide distributions, average read-depth and average sequence conservation.

Sequence context

We extracted strand specific sequence elements at the splice site (SS) and branch point regions from the human reference genome using BEDtools. We extracted 20 bp long sequences downstream of the 5' SS, upstream of the 3' SS and 10bp on either side of the BP for all non-overlapping introns. For the BP regions, all positions within 10 bp of the 3' SS were excluded to avoid effects on conservation analysis, etc. As a consequence, all introns with a BP to 3' SS distance < 10 bp were disregarded. We computed nucleotide (nt) frequencies using the "Biostrings" R package (54).
We used bits as a measure of the information content at each position in the sequences, calculated using Kulback-Leibler divergence, and visualized as logo plots. All negative values were disregarded, as we only considered nucleotide overrepresentation compared to our expectation and not depletion. In brief, Kulback-Leibler is a measure of divergence between the observed nucleotide frequencies at a position and the expected nucleotide distribution. Given a nucleotide *n* at a position *i*, the estimated divergence is given by

$$D_{KL}(p_i||q_i) = \sum_{n\in\{A,C,G,U\}} p_{n,i} \cdot \log_2\left(\frac{p_{n,i}}{q_{n,i}}\right)$$

Where $p_{n,i}$ is the observed nucleotide frequency at position *i* and $q_{n,i}$ is the expected frequency of *n* at *i*. To estimate $D_{KL}(p_i||q_i)$, we used a fixed q_n for all positions *i*. The expected discrete nucleotide distribution q_n is based on all non-host introns from non-host genes and is the total nucleotide frequency of the sequence of interest, i.e. 20 bp downstream of the 5'SS, 20 bp upstream of the 3' SS or 10 bp on either side of the BP.
Letter heights in the logo plot is proportional to the observed nt frequency and is defined as:

$$height_{n,i} = p_{n,i} \cdot D_{KL}(p_i||q_i)$$

with a bit being the sum of letter heights at a position. We used the "ggseqlogo" R package (55) to generate the Kulback-Leibler logo plots.

Intron read-depth

We found the position-wise read-depth for non-overlapping introns using BAM files containing mapped reads for all the NMIBC samples (n = 457). First, we only considered reads mapping directly to intronic loci and removed all linearly-spliced reads mapping across introns using their CIGAR strings and SAMtools "view". Then we utilized "BEDtools intersect" to select reads mapping at least partially to the non-overlapping intron annotations defined above. This step was strand-specific and only retained the read intersecting the intronic feature and not the whole read-pair. Second, a single bigWig file containing the aggregated read-depth from all samples was constructed. We achieved this by first generating individual bigWig files for each sample using DeepTools's bamCoverage (64) and then merging these files using UCSC's application bigWigMerge (65), which outputs a bedGraph file containing the read-depth at each intronic position summed over all NMIBC samples. We averaged the position-wise read-depths per sample. Then we sorted the bedGraph file and converted it to a bigWig file using the UCSC's application bedGraphToBigWig. The resulting bigWig file was used to visualize the read-depth in the IGV browser. Finally, we converted the bigWig file to a BED file format using "bwtools extract" (66) for further statistical analysis in R.
We used a similar approach to produce a bigWig file containing the read-depth of all non-filtered reads from the NMIBC samples.

Intron sequence conservation
We evaluated the intron sequence conservation using positional phyloP scores (67) from the 100-way vertebrate alignments downloaded from the UCSC Genome Browser (http://hgdownload.soe.ucsc.edu).
The intronic position-wise phyloP scores were extracted using “bwtool extract” and the outputted BED file used for analysis in R. The phyloP scores are based on multiple alignments between 100 vertebrate species and are measures of evolutionary conservation. PhyloP scores are -log(p-values) estimated under a null model of neutral evolution. Positive scores indicate conserved nucleotide positions, whereas negative values indicate fast-evolving sites (in comparison to expectation during neutral genetic drift) (67). We defined the whole intron conservation as the average position-wise conservation across the entire intron locus, with 10bp at splice site ends removed. Intron ends were removed due to the high conservation of splice site motifs creating a length bias. The length bias arises as a product of short introns having a larger percentage taken up by the universal and highly conserved SS motifs in comparison to longer introns.

SUPPLEMENTARY TABLE LEGENDS

Supp. Table S1. Primer sets used for RT-PCR. “Primers v1” were used in the RT-PCR run depicted in **Supplementary Figure S5** and “primers v2” were used for the results shown in **Figure 1I**.

Supp. Table S2. Annotations for the circular sisRNA junctions detected in non-muscle invasive bladder cancer (NMIBC). First columns indicate chromosomal position and host gene. The column “nr_collapsed_junctions” indicates how many junctions originally called by CIRCexplorer2 that were collapsed; “junction-spanning_reads” holds the summarized number of junction-spanning reads for the collapsed junctions; “nr_of_samples” contains the number of NMIBC tumor samples, from which junction-spanning reads are found; and “id_collapsed_junctions” has the id’s (chromosomal position) of the collapsed junctions. “cluster” indicates if the collapsed sisRNA junctions have a 3’ end distance larger than 10 bp (TRUE) or at most 10 bp (FALSE). The column “host_intron” indicates the genomic position of the intron hosting the stable circular sisRNA; “stable_lariat” whether the circular sisRNA is classified as a stable lariat (TRUE) or a 3’-5’ intronic circle (FALSE), and “length_bp” the length of the stable sisRNA given in base pairs (bp). The column “unique_intron_id” indicates the coordinates and gene name of the non-overlapping intron annotations. Finally, “detection_method” indicates which method successfully detected a given sisRNA cluster. All start positions are one-based. CE2 = CIRCexplorer2; CF = circRNA_Finder; CE2q = CE2-CIRIquant.

Supp. Table S3. Expression matrix of junction-spanning reads supporting predicted circular sisRNA junctions in the cohort of 457 patients with non-muscle invasive bladder cancer (NMIBC). For comprehensive circular sisRNA annotations see **Supplementary Table S2**. Note that the tables have matching rows.

Supp. Table S4. Normalized expression matrix of junction-spanning reads supporting predicted circular sisRNA junctions in the cohort of 457 patients with non-muscle invasive bladder cancer (NMIBC). The Expression is normalized for library size using reads per million (RPM). For comprehensive circular sisRNA annotations see **Supplementary Table S2**. Note that the tables have matching rows.

Supp. Table S5. Annotations for the circular sisRNA junctions detected in the ENCODE tissue data (n = 113). The first 14 columns are identical to **Supplementary Table S2**. Note, “nr_of_samples” indicate how many of the 113 samples (from the 46 tissues) that the circular sisRNA is expressed in. The additional 46 columns contain the mean normalized expression for the circular sisRNAs in each tissue type. The Expression is normalized for library size using reads per million (RPM).

Supp. Table S6. Table containing circular sisRNA summaries for different tissue types (ENCODE data).

Supp. Table S7. Summarizing tables of circular sisRNA properties in different cellular fractions of the bladder cancer cell lines FL3, HCV29 and T24 (**A**); the K562 cell line (**B**); and HepG2 (**C**).

SUPPLEMENTARY FILE LEGENDS

Supp. File S1. Bash script containing the code to extract all HepG2 total RNA-Seq samples from ENCODE.

Supp. File S2. Bash script containing the code to extract all K562 total RNA-Seq samples from ENCODE.

Supp. File S3. Bash script containing the code to extract all tissue total RNA-Seq samples from ENCODE.

SUPPLEMENTARY FIGURES

Supp. Figure S1

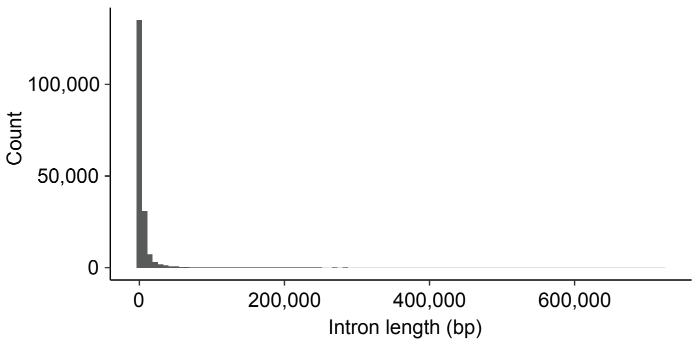

Supp. Figure S1. Intron length distribution. Intron length distribution of all non-overlapping introns (n = 183,203). bp = base pair.

Supp. Figure S2

A

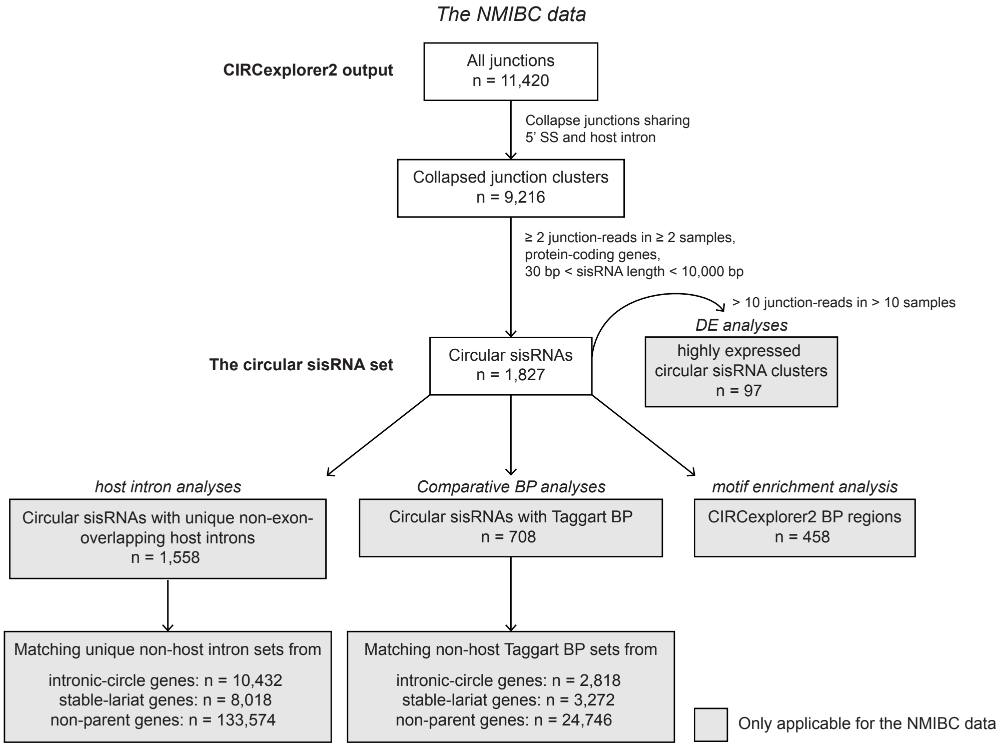

B

Data set	All junctions	Collapsed junction clusters	Circular sisRNA clusters	Read-support threshold
**NMIBC**	11,420	9,216	1,827	≥ 2 junction-reads in ≥ 2 samples
**RNase R**	19,287	13,188	342	≥ 2 junction-reads present in treated and control samples
**Tissue**	29,226	21,216	10,162	≥ 2 junction-reads in ≥ 1 samples
**BC cell lines**	244	160	149	None
**HepG2**	1,095	917	880	None
**K562**	8,889	6,172	5,630	None

Cell line data

Supp. Figure S2. Data-flow diagram and overview of circular sisRNA subsets. (**A**) Depiction of circular junction and circular sisRNA data subsets, including circular sisRNA sets with unique non-overlapping intron annotations, Taggart et al. BP annotations, and highly expressed circular sisRNAs. (**B**) Table depicting the number of detected circular junctions, circular-junction clusters, and circular sisRNA clusters as well as the read-threshold used for circular sisRNA detection. We only include circular sisRNAs derived from protein-coding genes and which have a length between 30-10 000 bp.

Supp. Figure S3

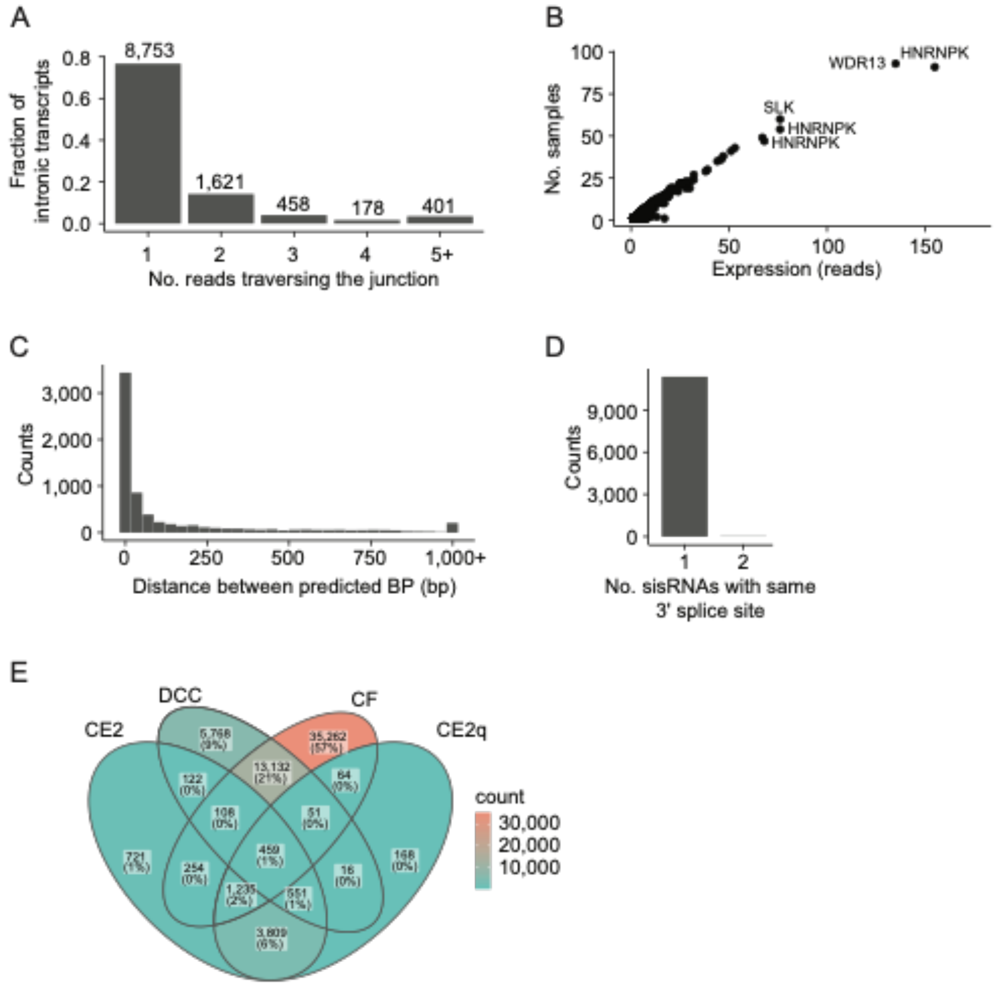

Supp. Figure S3. Identification and quantification of circular junctions. (**A**) Histogram depicting the number of junction-spanning reads supporting a predicted junction. (**B**) Summarized number of reads supporting a predicted junction plotted against the number of samples where the junction reads are expressed. (**C**) Distance between expected branch point coordinates for junctions that are annotated to the same 5' splice site. bp = base pair. (**D**) Number of junctions with the same 3' splice site but with different 5' start coordinates. (**E**) Venn diagram depicting the total number of overlapping junction clusters between the different circRNA detection methods; CIRCexplorer2 (CE2; n = 7335), DCC (n = 20 207), circRNA_Finder (CF; n = 50 565), and CE2-CIRIquant (CE2q; n = 6353). Due to several of the methods not adequately annotating junction origin (exonic or intronic), we defined intronic junctions as originating from the strictly non-exon overlapping intron loci.

Supp. Figure S4

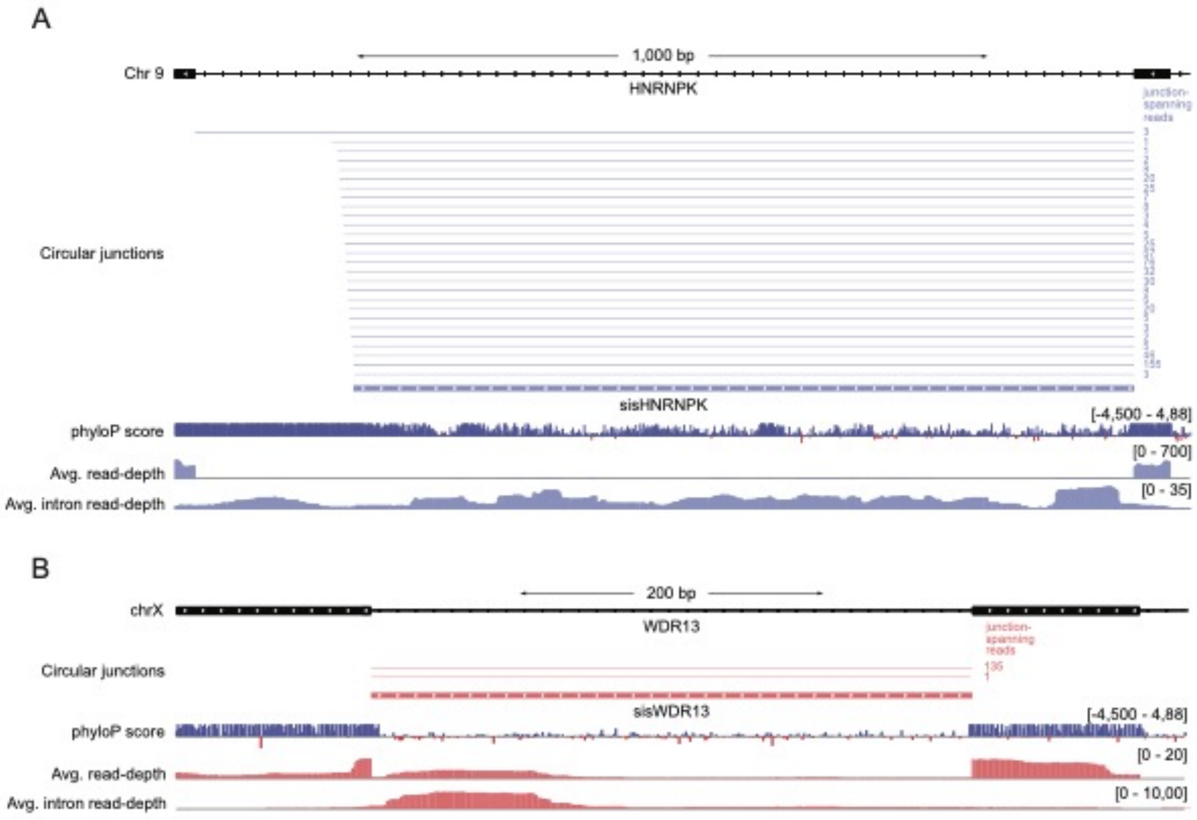

Supp. Figure S4. sisHNRNPK and sisWDR13 tracks. (**A**) The highly conserved intron from *HNRNPK* (chr9:83,975,506-83,976,994) harbors the circular sisRNA cluster sisHNRNPK. Depicted are the unique circular junctions constituting sisHNRNPK and their read support; the positional conservation (phyloP100Way scores); the average read-depth of linearly mapped reads across the NMIBC samples; and the average intronic read-depth of linearly mapped reads. (**B**) Overview of sisWDR13 derived from the second intron of *WDR13* (chrX:48,598,958-48,599,352). Includes the same tracks as depicted in panel A.

Supp. Figure S5

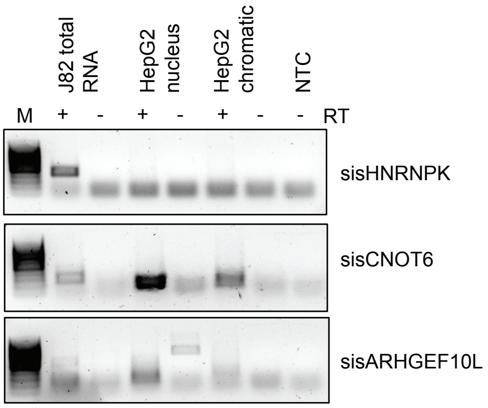

Supp. Figure S5. Circular sisRNA validation. RT-PCR results for select circular sisRNAs in the bladder cancer cell line J28 and fractionated HepG2 cells.

Supp. Figure S6

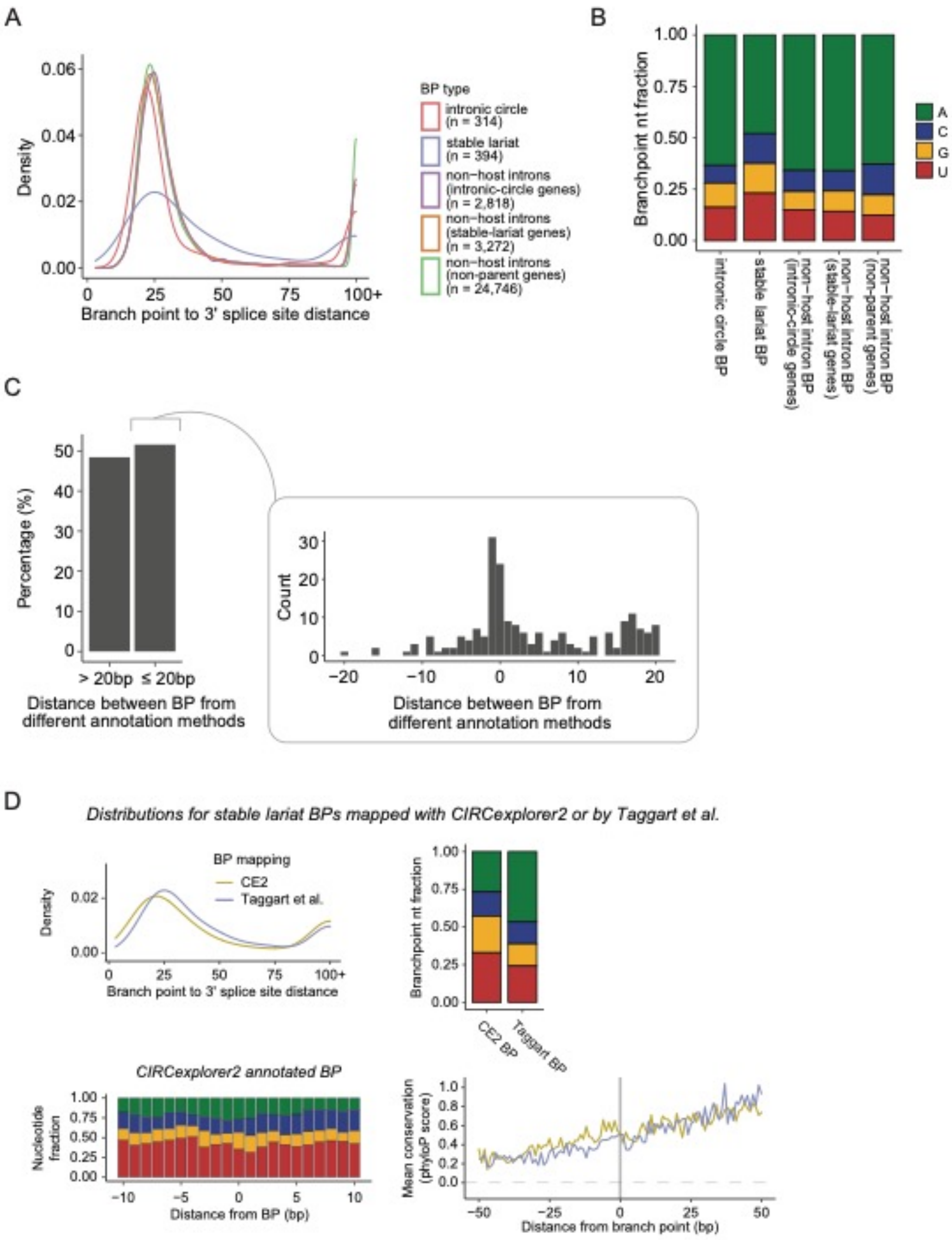

Supp. Figure S6. Characterization of the circular sisRNA branch point region. (**A**) Initial 3' tail length distributions (distance between predicted BP and 3' splice site). All intronic lariats with 3' tail lengths of 1bp or less are disregarded. Group colors indicate lariat class containing the BP used for computing the 3' tail length. (**B**) Nucleotide (nt) fractions at the branch point position for circular sisRNAs and conventional lariats. Same BP sets as used in panel A. A = adenine, U = uracil, G =

guanine, C = cytosine. (**C**) Distance between the Taggart et al. and CIRCexplorer2 annotated branch point positions for the stable lariats. (**D**) Comparison of BP-related properties given CIRCexplorer2 (CE2) and Taggart et al. (Taggart) annotated branch points for stable lariats. Top-left: Distance between predicted BP and 3' splice site; Top-right: Nucleotide fractions at the branch point position; Bottom-left: Nucleotide distribution at the BP locus based on CIRCexplorer2 BP annotations; Bottom-right: Mean positional conservation scores (phyloP100Way scores) for the 100 bases adjacent to the BP.

Supp. Figure S7

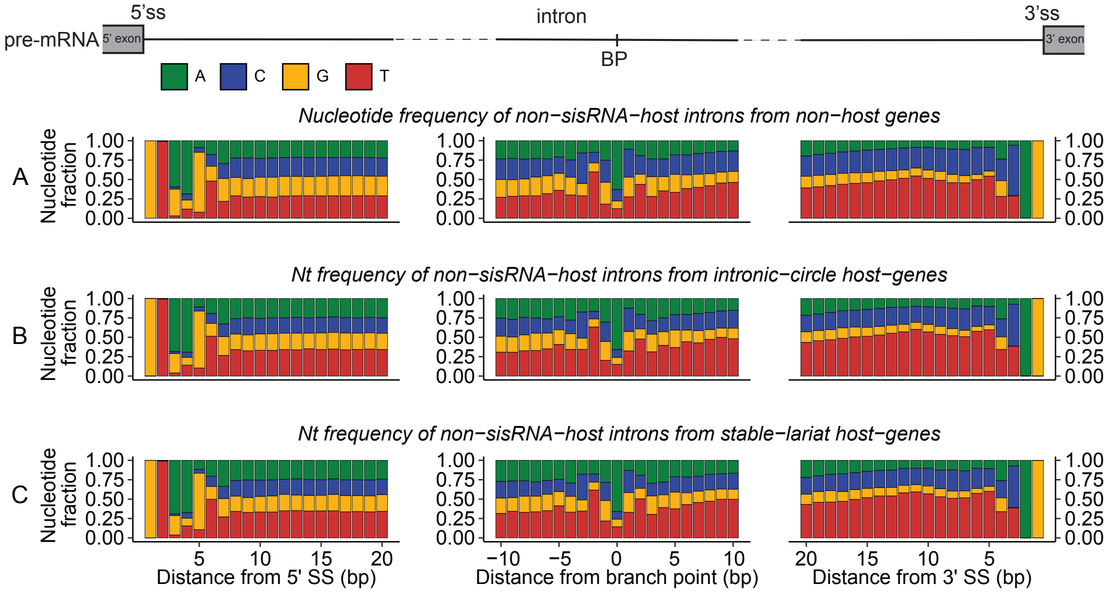

Supp. Figure S7. Positional nucleotide distributions of non-host introns. (**A-C**) Position-wise summaries based on sequence alignment at the 5’ SS (column one), BP (column two) and 3’ SS (column three). When considering the branch point region, ten nucleotides (nts) are removed from the intron ends to remove the splice site signal. A = adenine, U = uracil, G = guanine, C = cytosine. (**A**) Position-wise nucleotide distributions of introns from genes not hosting circular sisRNAs (includes 133 574 splice sites and 24 746 BP loci). (**B**) Positional nt distributions of non-host introns from genes hosting intronic circles (includes 10,432 splice sites and 2,818 BP loci). (**C**) Positional nt frequencies of non-host introns from genes hosting stable lariats (includes 8,018 splice sites and 3,272 BP loci).

Supp. Figure S8

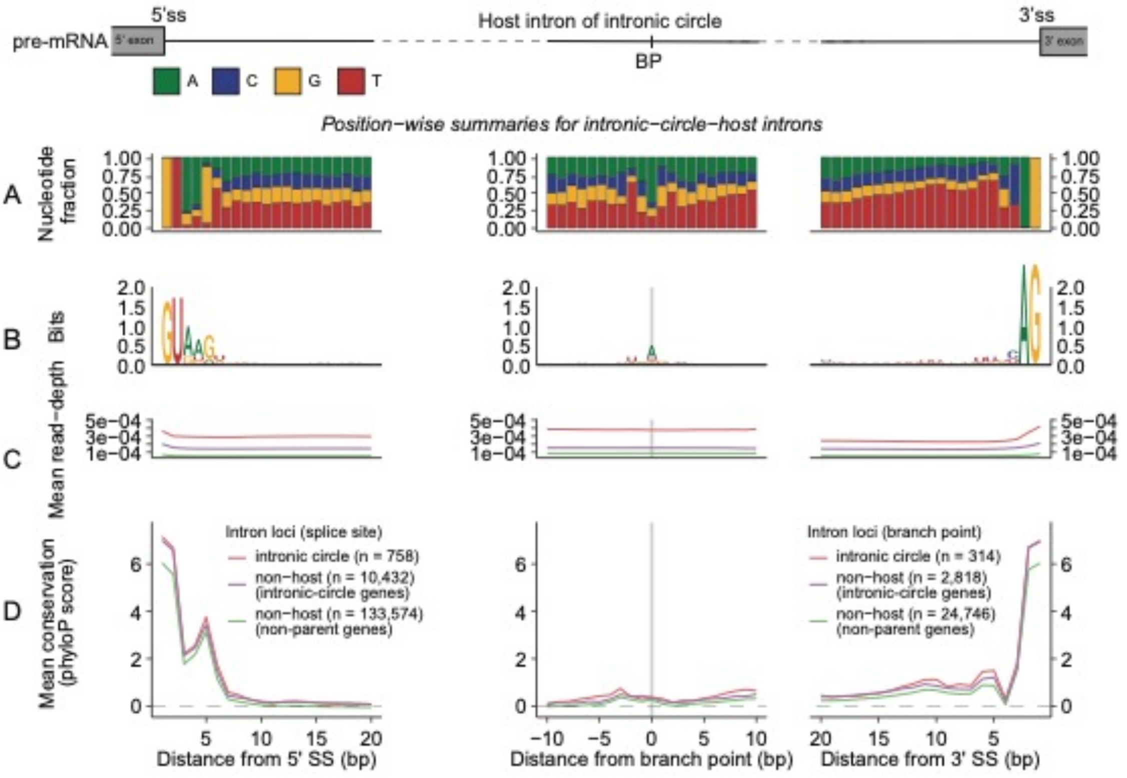

Supp. Figure S8. Position-wise characterization of intronic-circle host-intron sequences. (**A-D**) Position-wise summaries based on sequence alignment at the 5' SS (column one), BP (column two) and 3' SS (column three). When considering the branch point region, ten nts are removed from the intron ends to reduce the splice site signal. (**A**) Position-wise nucleotide frequencies of introns hosting intronic circles. A = adenine, U = uracil, G = guanine, C = cytosine. (**B**) Logo plots for 5' SS and 3' SS loci of intronic circles. (**C**) Mean position-wise read-depth found across the NMIBC samples. (**D**) Mean positional conservation scores (phyloP100Way score).

Supp. Figure S9

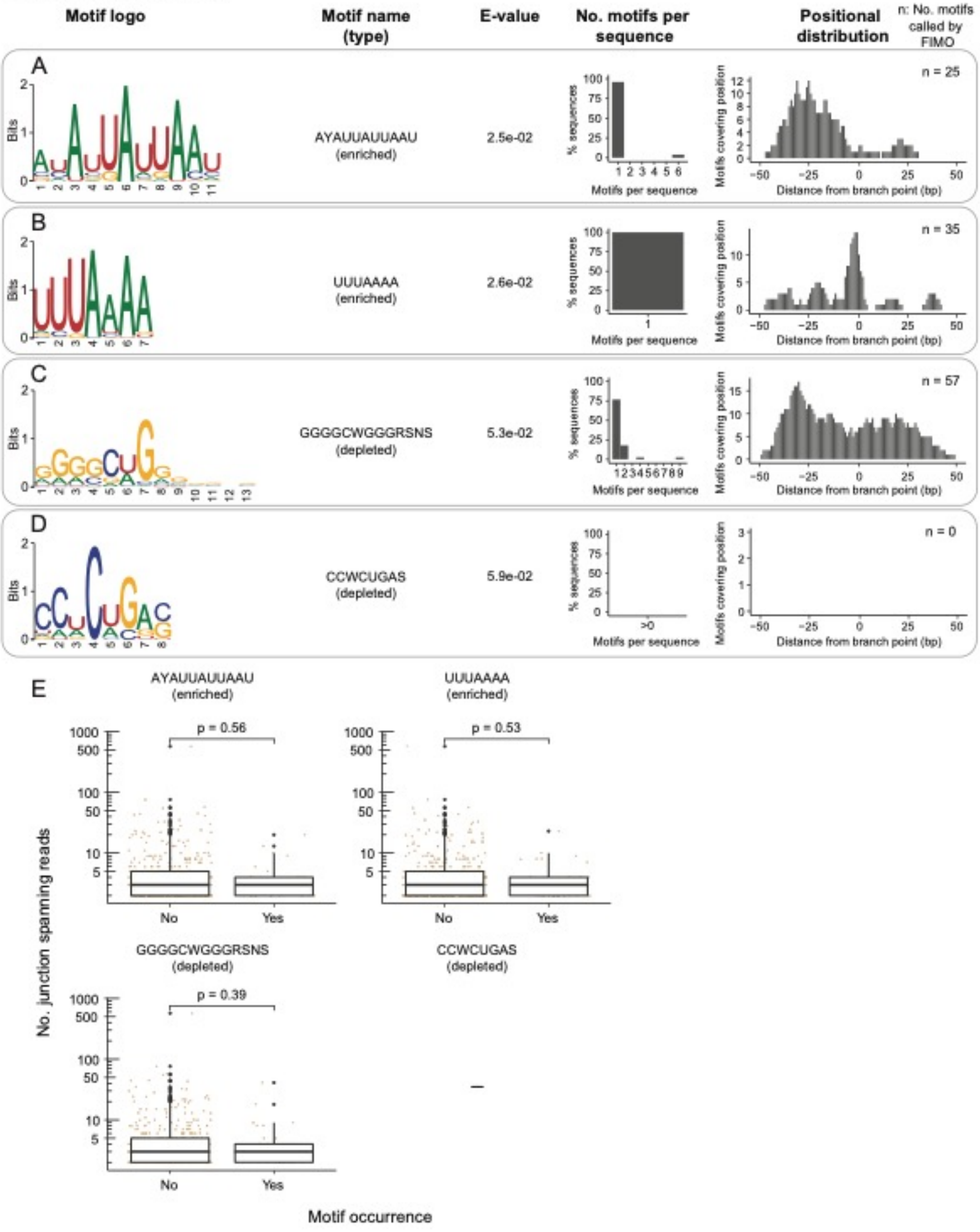

Supp. Figure S9. Catalog of significantly enriched or depleted motifs in the branch point locus of stable lariats. (**A-E**) A 100 bp region adjacent to the BP position is considered when performing de novo motif discovery analysis. (**A-D**) Motif overview, including sequence logo (column one); motif name and information regarding its enrichment state in stable lariats (column two); the level of significance of the enrichment/depletion (E-value < 0.1) (column three); motif occurrence and distribution across the branch point locus based on FIMO (Find motif occurrence) output (column four and five). Nucleotide and subset abbreviations: A (adenine), U (uracil), G (guanine), C (cytosine), W = {A, U}, B = {C, G, T}, Y = {C, U}, H = {A, C, T}, R = {A, G}, K = {G, T}, S = {C, G}. (**E**) Comparison of

stable lariat expression levels given motif occurrence. P-values are obtained by Wilcoxon rank-sum tests. The Y-axis is log10 scaled.

Supp. Figure S10

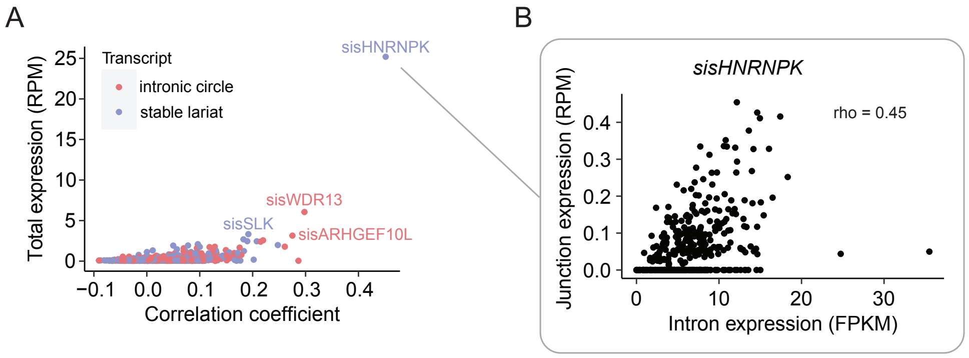

Supp. Figure S10. Expression correlation between circular sisRNA junctions and host introns. (**A**) Total circular sisRNA junction expression across all NMIBC samples plotted against the expression correlation of circular sisRNA and host intron (n = 1558). Spearman's correlation coefficient is used. Colors indicate type of transcript. RPM = reads per million. (**B**) Relationship between expression of sisHNRNPK and its host intron (chr9:83,975,506-83,976,994). Intron expression is normalized as fragments per kilobase million (FPKM) and circular sisRNA junctions as reads per million (RPM).

Supp. Figure S11

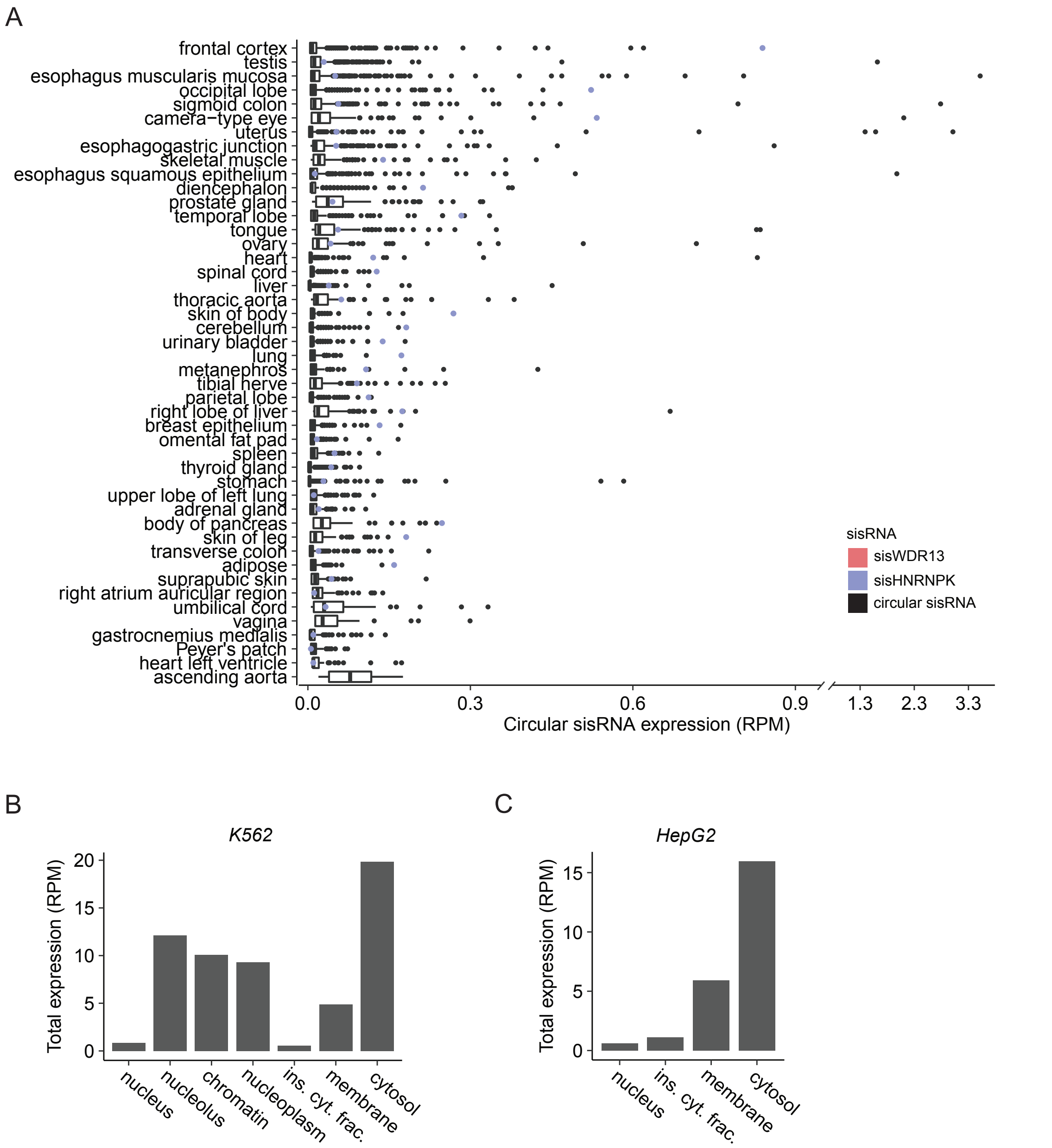

Supp. Figure S11. Circular sisRNA distribution across cell fractions and tissue types. (**A**) Expression levels of tissue-specific circular sisRNAs for each tissue type. Colors identify specific circular sisRNAs, with sisWDR13 being absent in all tissues. RPM = reads per million. (**B**) Overall expression of circular sisRNA in cell fractions of the K562 cell line. (**C**) Total circular sisRNA expression in HepG2 cell fractions.

Supp. Figure S12

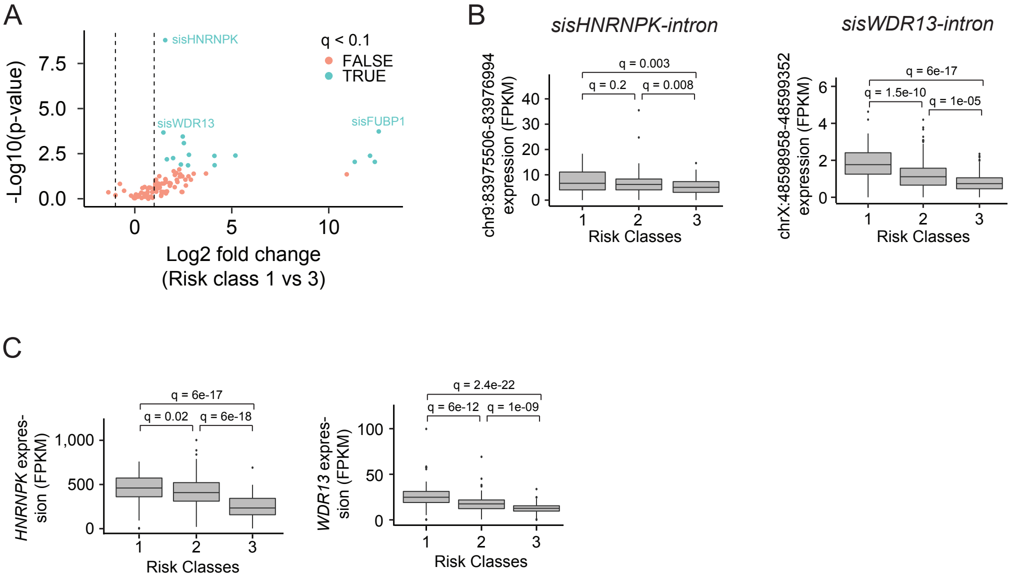

Supp. Figure S12. sisHNRNPK and sisWDR13 expressions are correlated with clinical outcomes. (**A**) Differential expression analysis of circular sisRNAs between risk class 1 (n = 96) and 3 (n = 129) tumors from patients with NMIBC. The $\log_2$ fold changes (class 1 vs class 3) are plotted against the negative $\log_{10}$(p-values). Colors indicate if genes are significantly (blue) or not significantly (red) differentially expressed after Benjamini-Hochberg FDR control (q < 0.1). Vertical lines indicate a log FC > 1 or < -1. (**B+C**) Expression of the entire host introns of sisHNRNPK (B, left) and sisWDR13 (B, right) as well as the parent genes *HNRNPK* (C, left) and *WDR13* (C, right) in risk class 1, 2, and 3 tumors. q-values are obtained from adjusted Wilcoxon rank-sum tests using BH FDR control.

Supp. Figure S13

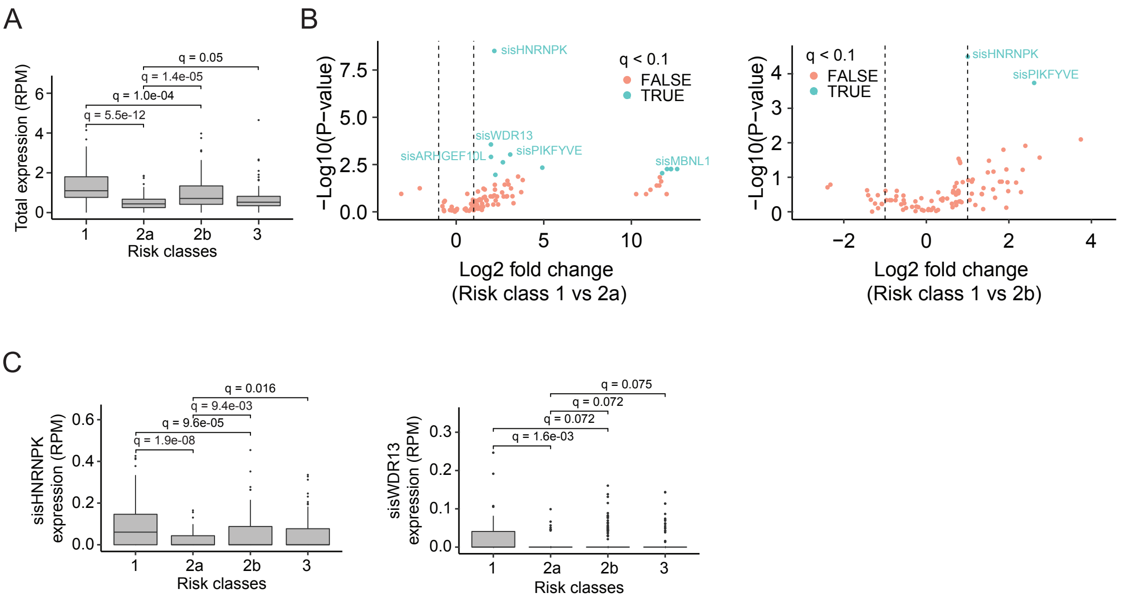

Supp. Figure S13. Circular sisRNA expression across expanded prognostic risk classes. (**A**) The total NMIBC tumor expression of circular sisRNAs across the expanded prognostic risk classes. RPM = reads per million. (**B**) Differential expression analysis of highly abundant circular sisRNAs (more than ten reads across ten samples). Left: Comparison of risk class 1 (n = 101) and 2a (n = 84), with 12 cases found to be significant. Right: Comparison of risk class 1 (n = 101) and 2b (n = 169), with two significant cases. Blue points indicate circular sisRNAs significant after BH FDR control. Vertical lines indicate a log FC > 1 or < -1. (**C**) sisHNRNPK (left) and sisWDR13 (right) expression across the expanded prognostic risk classes. q-values were obtained by BH FDR control.

Supp. Figure S14

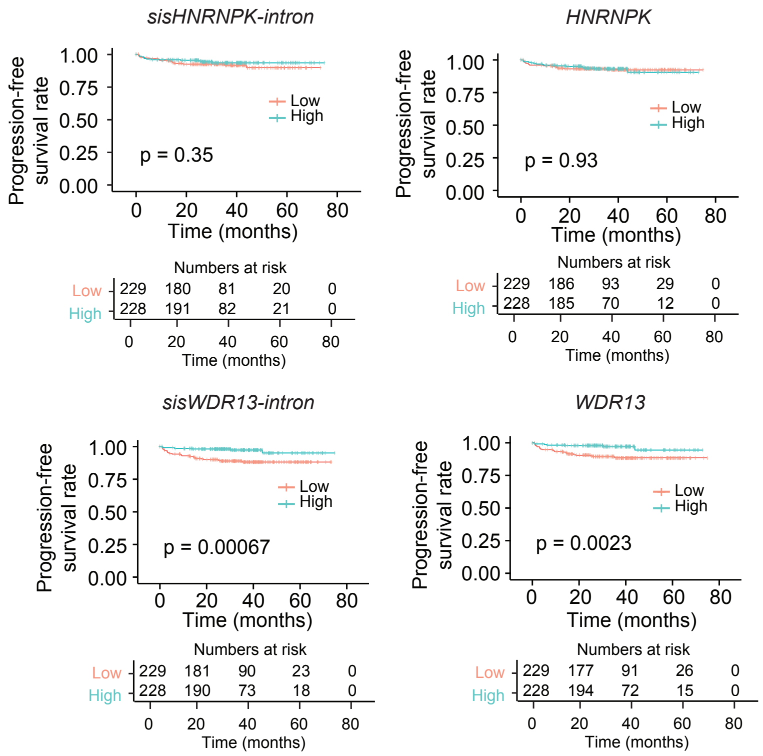

Supp. Figure S14. Progression free survival correlations for host introns and parent genes of sisHNRNPK and sisWDR13. Kaplan-Meier progression-free survival plots for the entire host introns of sisHNRNPK (top, left) and sisWDR13 (bottom, left) as well as the parent genes *HNRNPK* (top, right) and *WDR13* (bottom, right) in patients with NMIBC. Median expression used as cut-off; sisHNRNPK-intron = 5.98 FPKM, sisWDR13-intron = 1.09 FPKM, *HNRNPK* = 375 FPKM, and *WDR13* = 17 FPKM. P-values were obtained by Log-Rank Test.
