# Supplementary material for: Circular stable intronic RNAs possess distinct biological features and are deregulated in bladder cancer
Source: NAR Cancer. 2023 Aug 7;5(3):zcad041. doi: 10.1093/narcan/zcad041 (PMC10405568; doi:10.1093/narcan/zcad041)
Supplement: zcad041_Supplemental_Files [file zcad041_supplemental_files.zip › Table_S1_Primers_sets.pdf]

Supp. Table S1

Primers v1

sisHNRNPK FW	GAGCCAGCCTGCTACTGAAA
sisHNRNPK RE	CCCTGCTCAATCTGGGCTTT
sisWDR13 FW	TCAAGCACCTGCTCTTTGC
sisWDR13 RE	GAATGTGGCCAGAAGCTCA
sisARHGEF10L FW	ATGAGGGCAGGTAGGGAGAG
sisARHGEF10L RE	TCCCCATAAAGCAGTGCTGG
sisCNOT6 FW	GGATGTGGGAAGCTTGATGC
sisCNOT6 RE	TAGTTTCCACACCCTTGGCC

Primers v2

sisHNRNPK FW	GAGCCAGCCTGCTACTGAAA
sisHNRNPK RE	CCCTGCTCAATCTGGGCTTT
sisWDR13_v2 FW	TCAGGGCTCCACACCTCAA
sisWDR13_v2 RE	CAGTGGCTTCCTCACGAGTT
sisARHGEF10L_v2 FW	TAATTGGAGAGTGACGCGGG
sisARHGEF10L_v2 RE	GCCCACAGTCCCCAAACA
sisCNOT6_v2 FW	CCCTCTATGACGGTACAGGGA
sisCNOT6_v2 RE	TTTCCACACCCTTGGCCC
