# Supplementary material for: Circular stable intronic RNAs possess distinct biological features and are deregulated in bladder cancer
Source: NAR Cancer. 2023 Aug 7;5(3):zcad041. doi: 10.1093/narcan/zcad041 (PMC10405568; doi:10.1093/narcan/zcad041)
Supplement: zcad041_Supplemental_Files [file zcad041_supplemental_files.zip › Table_S6_circular_sisRNA_tissue_summaries.pdf]

Supp. Table S6

	# circular sisRNAs	% intronic circles	mean expression (RPM)	sd	median length (bp)	IQR	average library size
frontal cortex	1690	14.8	0.016	0.040	706	935	2.55e+08
testis	1302	1.5	0.037	0.084	444	596	8.76e+07
esophagus muscularis mucosa	928	3.3	0.096	0.312	338	319	4.93e+07
occipital lobe	1209	14.6	0.016	0.031	735	920	2.03e+08
sigmoid colon	660	5.6	0.106	0.289	359	343	4.46e+07
camera−type eye	422	8.1	0.036	0.111	653	840	1.46e+08
uterus	831	4.7	0.054	0.393	511	727	9.19e+07
esophagogastric junction	612	2.3	0.095	0.138	390	479	4.77e+07
skeletal muscle	519	7.5	0.027	0.038	450	727	1.94e+08
esophagus squamous epithelium	633	3.0	0.082	0.234	507	897	4.81e+07
diencephalon	759	14.2	0.016	0.029	519	774	1.46e+08
prostate gland	250	3.2	0.094	0.098	455	561	5.23e+07
temporal lobe	589	15.3	0.020	0.032	660	922	1.73e+08
tongue	230	7.8	0.048	0.089	515	832	1.44e+08
ovary	240	4.6	0.067	0.102	408	548	5.92e+07
heart	1071	7.4	0.014	0.048	613	919	1.65e+08
spinal cord	721	11.7	0.010	0.011	535	867	2.13e+08
liver	1274	4.8	0.010	0.024	595	1083	2.64e+08
thoracic aorta	215	5.6	0.061	0.092	655	1015	7.10e+07
skin of body	599	8.0	0.011	0.016	522	919	1.93e+08
cerebellum	684	11.4	0.009	0.013	499	859	2.09e+08
urinary bladder	599	5.7	0.010	0.013	383	740	2.17e+08
lung	524	6.9	0.011	0.011	520	851	2.32e+08
metanephros	433	5.8	0.013	0.026	286	617	1.95e+08
tibial nerve	276	6.5	0.084	0.104	368	425	5.94e+07
parietal lobe	651	14.4	0.008	0.011	497	822	2.05e+08
right lobe of liver	152	7.2	0.070	0.101	521	835	4.70e+07
breast epithelium	522	8.4	0.030	0.029	529	781	7.47e+07
omental fat pad	519	3.5	0.038	0.028	589	869	4.73e+07
spleen	385	7.0	0.051	0.045	705	923	4.04e+07
thyroid gland	983	7.9	0.024	0.033	539	826	1.00e+08
stomach	400	6.5	0.057	0.230	439	782	7.36e+07
upper lobe of left lung	337	5.9	0.053	0.055	549	934	4.65e+07
adrenal gland	370	4.1	0.047	0.046	520	750	6.85e+07
body of pancreas	94	4.3	0.089	0.103	665	1113	4.84e+07
skin of leg	210	10.0	0.079	0.085	516	665	5.32e+07
transverse colon	399	5.0	0.039	0.076	463	825	7.23e+07
adipose	339	9.1	0.045	0.039	537	777	5.00e+07
suprapubic skin	255	6.3	0.059	0.053	566	916	4.09e+07
right atrium auricular region	160	7.5	0.043	0.033	520	610	4.96e+07
umbilical cord	62	0.0	0.051	0.065	519	980	1.83e+08
vagina	49	2.0	0.050	0.055	318	322	7.35e+07
gastrocnemius medialis	213	6.1	0.045	0.056	485	580	6.68e+07
Peyer's patch	159	2.5	0.052	0.041	601	1026	4.08e+07
heart left ventricle	99	9.1	0.040	0.039	534	578	5.15e+07
ascending aorta	5	20.0	0.086	0.063	968	3405	5.13e+07
