# Supplementary material for: Circular stable intronic RNAs possess distinct biological features and are deregulated in bladder cancer
Source: NAR Cancer. 2023 Aug 7;5(3):zcad041. doi: 10.1093/narcan/zcad041 (PMC10405568; doi:10.1093/narcan/zcad041)
Supplement: zcad041_Supplemental_Files [file zcad041_supplemental_files.zip › Table_S7_circular_sisRNA_CL_Frac_summaries.pdf]

Supp. Table S7

A

	compartment	# circular sisRNAs	% intronic circles	mean expression (RPM)	sd	median length (bp)	IQR	library size
FL3	*cytoplasm*	20	15.0	0.038	0.011	714	997	2.92e+07
	nucleus	8	0.0	0.045	0.014	1021	1157	2.49e+07
HCV29	*cytoplasm*	6	50.0	0.090	0.050	637	569	1.66e+07
	nucleus	2	50.0	0.046	0.000	891	233	2.20e+07
T24	*cytoplasm*	70	15.7	0.012	0.005	1047	1569	9.43e+07
	nucleus	49	18.4	0.012	0.005	619	1055	9.35e+07

B

K562	# circular sisRNAs	% intronic circles	mean expression (RPM)	sd	median length (bp)	IQR	library size
chromatin	2198	0.5	0.005	0.003	587	1007	2.62e+08
nucleolus	1640	0.5	0.007	0.043	510	871	2.19e+08
nucleoplasm	1801	0.8	0.005	0.008	422	773	2.60e+08
membrane	215	0.5	0.023	0.015	840	1009	5.66e+07
nucleus	39	0.0	0.021	0.005	419	544	4.96e+07
insoluble cytoplasmic fraction	28	0.0	0.020	0.007	1176	1746	5.49e+07
cytosol	523	0.6	0.038	0.063	403	420	5.25e+07

C

HepG2	# circular sisRNAs	% intronic circles	mean expression (RPM)	sd	median length (bp)	IQR	library size
nucleus	41	0.0	0.015	0.006	846	1281.00	7.31e+07
insoluble cytoplasmic fraction	105	1.9	0.011	0.005	706	1491.00	1.12e+08
cytosol	544	0.7	0.029	0.070	538	578.25	6.31e+07
membrane	321	0.9	0.018	0.015	1087	1167.00	7.10e+07
